# Halide Perovskites for Supercapacitors and Photosupercapacitors: Recent Developments and Future Perspectives

**DOI:** 10.1002/smll.202503138

**Published:** 2025-06-17

**Authors:** Chandra Sekhar Rout, Pratik Shinde, Mohammed Arkham Belgami, Jung Sang Cho, Sang Mun Jeong

**Affiliations:** ^1^ Centre for Nano and Material Sciences Jain (Deemed–to–be University) Jain Global Campus Kanakapura Road Bangalore Karnataka 562112 India; ^2^ Department of Chemical Engineering Chungbuk National University Cheongju Chungbuk 28644 Republic of Korea; ^3^ Department of Molecular Sciences and Nanosystems Ca’ Foscari University of Venice Via Torino 155 Venice Venezia 30172 Italy; ^4^ Department of Engineering Chemistry Chungbuk National University Cheongju Chungbuk 28644 Republic of Korea; ^5^ Advanced Energy Research Institute Chungbuk National University Cheongju Chungbuk 28644 Republic of Korea

**Keywords:** efficiency, perovskite, photosupercapacitor, solar cells, stability, supercapacitor

## Abstract

Supercapacitors have garnered significant research interest as high‐performance energy storage devices, owingto their high power density and excellent recyclability. Advances in efficiencyand flexibility have led to the development of photosupercapacitors, whichstore charge electrochemically using solar energy. The performance andefficiency of a photosupercapacitor are primarily determined by the materialsused in its fabrication. Halide perovskites have become a popular material forsupercapacitors and photosupercapacitors, because of their high crystallinity, charge storage capacity, electrochemical properties, ease of synthesis, andcost‐effectiveness. Although challenges remain in the reproducibility, reliability, and long‐term performance of their devices. This article aims topresent a comprehensive review of the applications and advancements of halideperovskites in supercapacitors and photosupercapacitors. Initially, thestructural aspects of halide perovskites are introduced, and then the workingprinciples and insights of both applications are briefly discussed.Subsequently, the progress of halide perovskites in supercapacitors andphotosupercapacitors is thoroughly discussed. Likewise, the limitationsassociated with the perovskite‐based supercapacitors and photosupercapacitorsand their progress achieved to overcome the limitations have been discussed. Inthe end, current challenges associated with halide perovskites, theirsupercapacitor devices, possible solutions, and future perspectives are summarised.

## Graphical abstract

This article provides a comprehensive review of supercapacitor and photosupercapacitor applications of halide perovskites and their future perspectives.

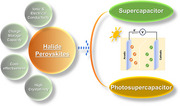

## 1 Introduction

Supercapacitors represent alternative electrochemical energy storage devices to address the huge need for renewable energy sources.^[^ ^1^, ^2^, ^3^, ^4^, ^5^, ^6^ ^]^ Research on the development of high‐performance supercapacitors has gained tremendous interest in recent years because of their high power density (>10 kW Kg^−1^), long cycle life (>10^5^ cycles) and bridged function of the power/energy gap between traditional dielectric capacitors (high power density) and batteries (high energy density).^[^ ^7^, ^8^, ^9^, ^10^ ^]^ In contrast to batteries, supercapacitors have a low energy density, which hinders their potential for commercialization. Therefore, it is crucial to design supercapacitors with high energy density while retaining the high‐power density. Since, the energy density, *E* = ½ CV^2^, the energy density can be upgraded by improving either capacitance or potential window value. To produce high‐performance supercapacitors, it is crucial to develop suitable electrode materials and electrolytes. In this regard, different electrode materials with good electrical/ionic conductivity, porosity, high surface area, flexibility, and tunable physicochemical properties are being investigated. Different electrode materials based on carbon and its derivatives, transition metal oxides/chalcogenides, MXenes, conducting polymers, black phosphorus, etc. have emerged as promising candidates to achieve the requirement.^[^ ^1^, ^2^, ^3^, ^4^, ^5^ ^]^ Further, different innovative approaches such as device miniaturization, modification of the electrode materials by doping, alloying, hybridizing, defect/vacancy engineering, etc. are being employed to further improve the performance.

**Scheme 1:**
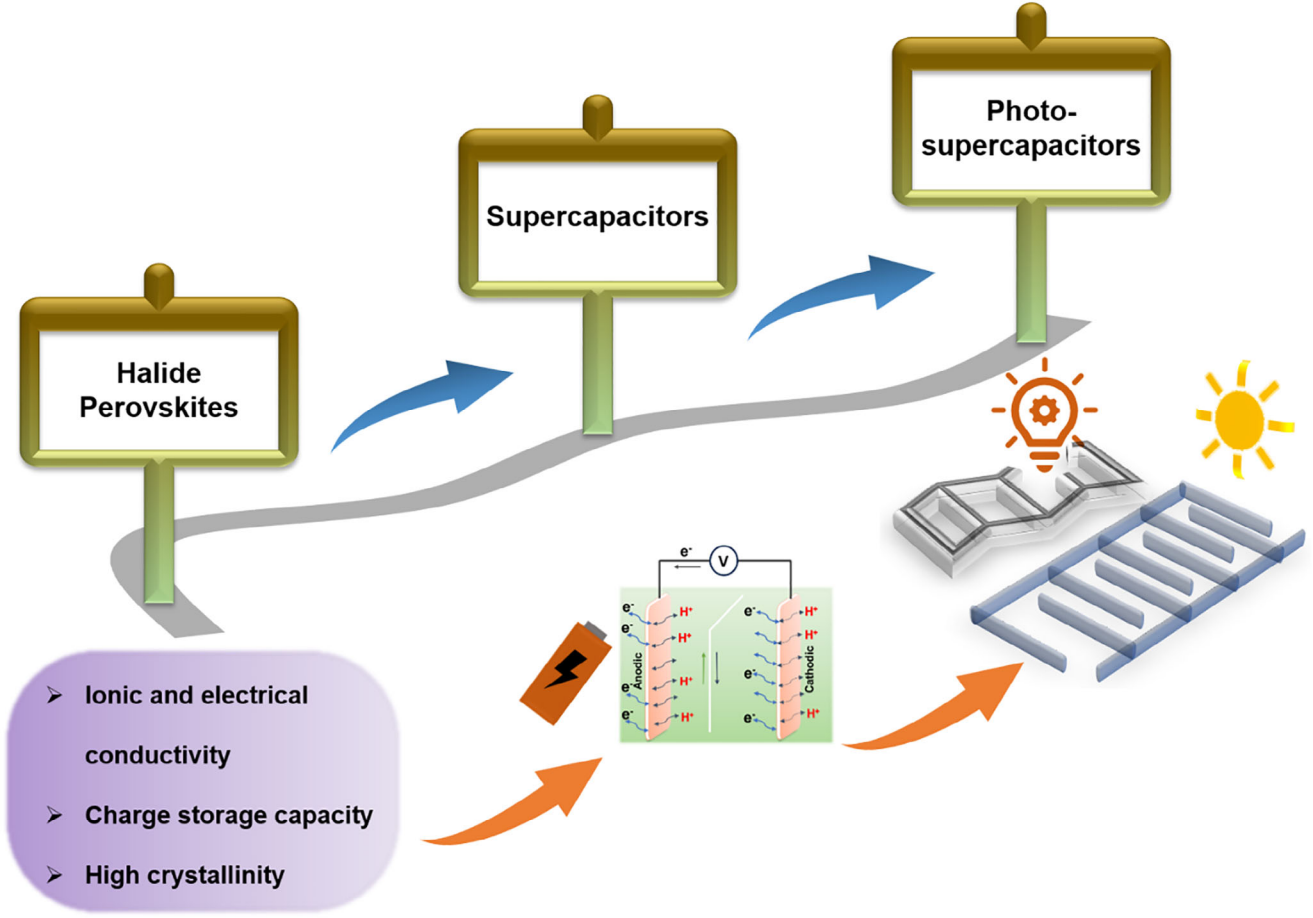
Progress of halide perovskite materials for supercapacitor and photosupercapacitor applications.

Halide Perovskites have gained much attention since their discovery in 2009 due to their excellent optoelectronic properties, leading to high‐performance solar cells with superior power conversion efficiency (≈26%).^[^ ^10^, ^11^, ^12^, ^13^, ^14^ ^]^ Additionally, these materials have been described as highly appealing candidates for ferroelectric, nonlinear optical, and birefringent materials.^[^ ^15^, ^16^, ^17^ ^]^ Halide perovskites are recognized as promising candidates for supercapacitors because of their admirable ionic and electrical conductivity, high crystallinity, good electrochemical properties and charge storage capacity, easy preparation and cost‐effectiveness. Further, the presence or creation of vacancies in perovskites promotes their suitability for supercapacitor applications since it facilitates ionic migration, ionic conductivity and yet increases the surface area.^[^ ^18^ ^]^ The integrated solar cells and supercapacitors known as photosupercapacitors, based on halide perovskites have drawn much attention in recent years.^[^ ^19^ ^]^ **Scheme** **1** provides information on the roadmaps of the research developments on supercapacitors and photosupercapacitors based on halide perovskites. In this review, we discuss the recent progress on halide perovskites‐based supercapacitors and photosupercapacitors. Further, we brief information on the halide perovskites, their importance for supercapacitors, likewise, the limitations associated with the perovskite‐based supercapacitors and supercapacitors and the progress achieved to overcome the limitations have been discussed. Finally, the challenges, opportunities, and future perspectives of this research field are comprehensively analyzed.

## 2 Background

### 2.1 Types and Crystal Structure of Halide Perovskites

**Figure 1:**
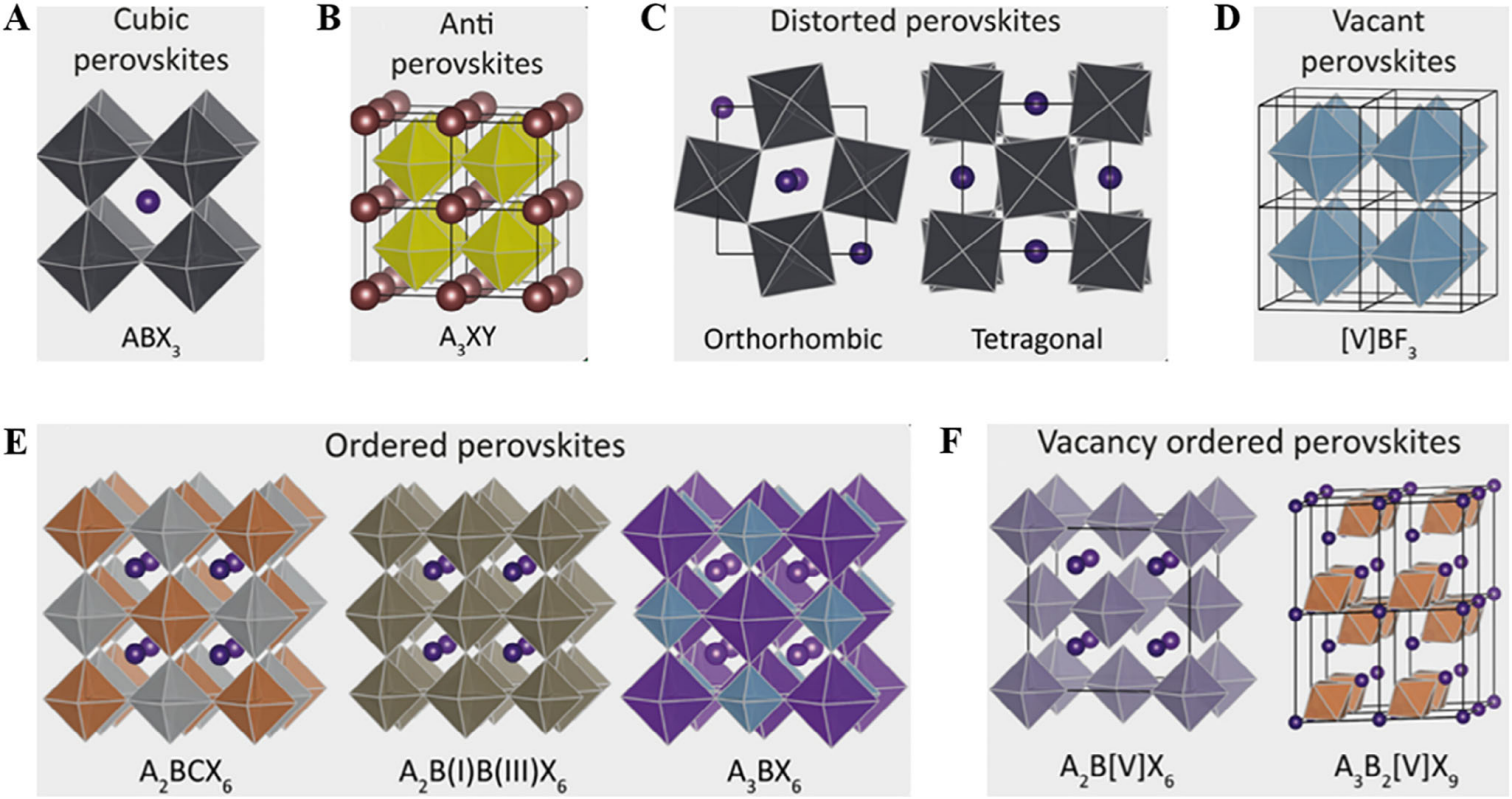
A) ABX_3_ cubic halide perovskites B) Antiperovskites, with A being a monovalent metal (like Li^+^ or Ag^+^), X a halide, and Y a chalcogenide C) Structure of orthorhombic and tetragonal disordered perovskites, formed due to tilt in octahedra D) Vacant BX_3_ perovskites, (such as AlF_3_) E) Structures of ordered perovskites, where two M(II) metals are changed by a M(I) and M(III) metal F) Structures of vacancy ordered perovskites, in this structure B‐site cations is replaced with a M(III) or M(IV) and vacancies. Adapted with permission.^[^ ^20^ ^]^ Copyright 2020, American Chemical Society.

Perovskites belong to a general ABX_3_ type crystal with a network of corner‐sharing BX_3_ octahedra and have *Pm* $\bar{3}$ *m* space group.^[^ ^8^, ^20^ ^]^ However, the formation of double or quadruple perovskites can occur due to the deviation from the ABX_3_ stoichiometry through the vacancy formation at A or B cation sites or by replacement of the cations with other atoms. By following these modifications, perovskites can exist in four different dimensions (0D, 1D, 2D, and 3D) with the forms of (i) ABX_3_, (ii) A_2_BX_4_ (layered perovskites), (iii) A_2_BB'X_6_ (double perovskites), and (iv) A_2_A'B_2_B'X_9_ (double perovskites).^[^ ^10^, ^21^ ^]^ Due to the full or partial substitution and replacement of cations/anions, perovskites show tunable optical and electronic properties, which make them ideal candidates for different applications. CaTiO_3_ mineral perovskite was first discovered by Gustav Rose in 1839, later on, Alekseyevich von Peroski, characterized it.^[^ ^21^ ^]^ Due to the distortions from its cubic crystals, perovskites also exhibit other crystal structures such as orthorhombic, tetragonal and rhombohedral (**Figure** **1**A–F).^[^ ^20^ ^]^ In the case of metal halide perovskites, the B cations are divalent (Ex: Pb^2+^ or Sn^2+^) and A cations are large monovalent alkali metals (Ex: Cs) or small organic cations such as methyl‐ammonium (MA) or formamidinium (FA). The stability of the perovskites depends on the ionic size of the cations and anions at the A, B, and X sites and can be determined by the “tolerance factor,”

(1) $$t=\frac{r_{A}+r_{X}}{\sqrt{2}\left(r_{B}+r_{X}\right)}$$

where *r_A_, r_B,_* and *r_X_* are the ionic radii of *A*, *B* and *X* ions, respectively. Hexagonal, orthorhombic, rhombohedral, and cubic structures of perovskites have respective t values as 1.00 < *t* < 1.13, 0.9 < *t* < 1.0, 0.75 < *t* < 0.9, and *t* = 1, respectively. Halide antiperovskites also exist when A and B sites are occupied by the anions (halide and chalcogenides) and X sites are occupied by the monovalent cation (Ex: Li_3_OBr, Ag_3_SI) (Figure 1B).^[^ ^22^ ^]^ A site vacant BX_3_ crystal can exist when the A cation site is fully vacant in a perovskite (Ex: AlF_3_, FeF_3_, MnF_3_, and CoF_3_) (Figure 1D). Figure 1E shows the crystal structures of ordered double perovskites in which the B cations are heterovalently replaced by a combination of two (or more) cations. This kind of structure is termed as A_2_BCX_6_ or A_2_BB'X_6_ elpasolite structure (Ex: Cs_2_AgInCl_6_, Cs_2_AgBiBr_6_, MA_2_AgSbI_6_, etc.). Some groups of ordered perovskites also crystallize in the A_3_BX_6_ (or A_2_ABX_6_) cryolite phase where half of the B sites are occupied by the cations “A” (Ex: Na_3_AlF_6_) (Figure 1E). When the B‐site cation is partially replaced with a vacancy, they are called vacancy‐ordered perovskites (Cs_2_SnI_6_, Cs_2_PdBr_6_, Cs_2_TiBr_6_, Cs_2_TeI_6_, Cs_3_Sb_2_I_9_ etc.) (Figure 1F).

### 2.2 Supercapacitors

**Figure 2:**
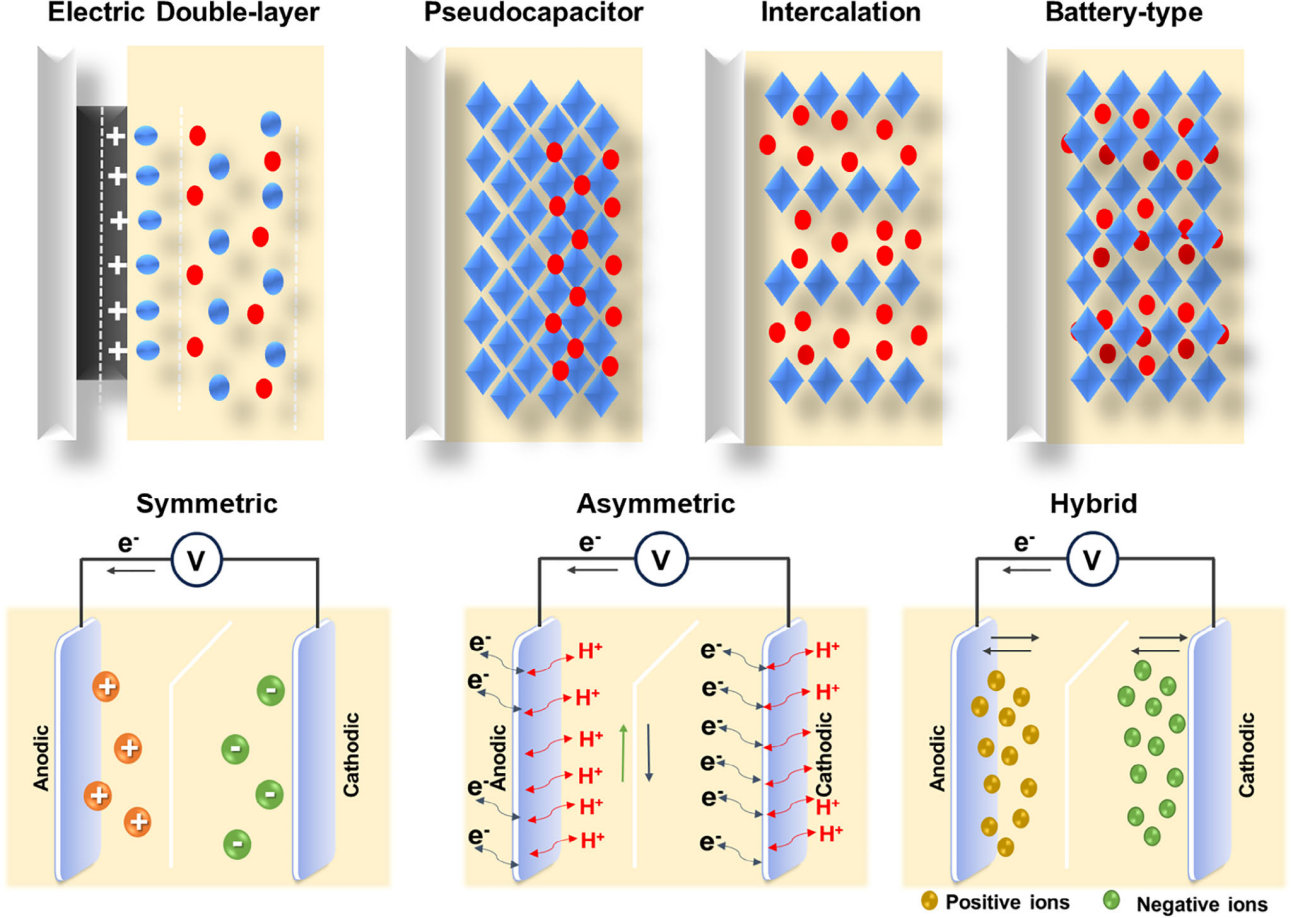
Different types of supercapacitors describing EDLC, pseudocapacitor, intercalation, and battery type.

**Figure 3:**
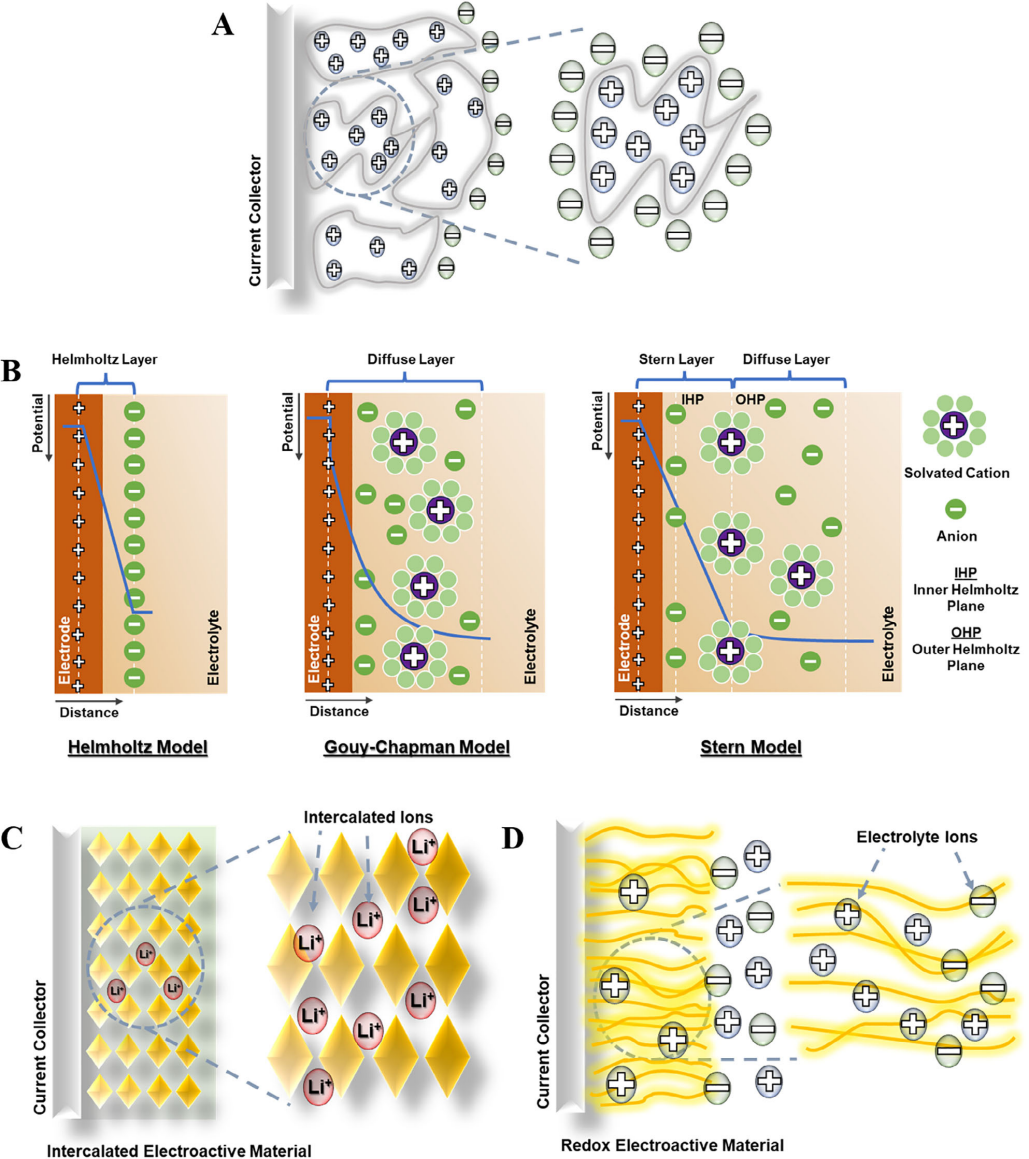
A) Schematic illustration of porous carbon electrode storage mechanism B) Representation of different EDLC models such as Helmholtz model, Gouy–Chapman model, and Gouy–Chapman–Stern model C) intercalation mechanism D) surface redox mechanism.

Supercapacitors can be classified into three main types based on their different charge storage processes: electric double‐layer capacitors (EDLC), pseudocapacitors, and hybrid supercapacitors (**Figure** **2**).^[^ ^23^, ^24^, ^25^ ^]^ In the case of EDLCs (mostly for carbon‐based electrode materials), charge storage occurs due to the generation of an electric double layer at the porous electrodes and electrolyte interface and involves a non‐Faradaic process. In electrolytes, cations propel toward the negative electrode whereas the anions move toward the positive electrode during the charging process, whereas the reverse process occurs during discharging (Figure 2 and **Figure** **3**A). Since there is no involvement of any Faradaic reaction during these processes, the concentration of the electrolyte ions remains the same and the electrode materials retain their crystallinity, resulting in high cycling stability up to millions of cycles. This phenomenon was first proposed by Hermann von Helmholtz in 1853 (Figure 3B). As per the model, the so formed double layer at the interface of the electrode and electrolyte is known as the Helmholtz layer.^[^ ^26^, ^27^ ^]^ Guoy and Chapman modified this model by introducing a diffusion layer since it is formed due to the thermal motion of ions in the electrolyte (Figure 3B). Since this model failed for more highly charged electrodes or high concentrations of ions, Stern projected a model by merging both Helmholtz and, Guoy‐Chapman models. In this model, the Stern/compact layer was divided into the inner Helmholtz plane (IHP) and the outer Helmholtz plane (OHP) (Figure 3B). In pseudocapacitors, the charge storage process occurs as a result of the Faradaic redox reactions at or near the electrode surface following the electrochemical adsorption/desorption or intercalation/de‐intercalation of the ions into the tunnels of the active materials (Figure 3C–D).^[^ ^23^, ^24^ ^]^ Pseudocapacitors deliver high energy density and capacitance values compared to the EDLC‐type supercapacitors due to the Faradaic process and surface redox reactions. In the case of the intercalation process, the ions tunnel through the layers of the active electrode materials during the Faradaic transfer and it is mostly observed for the 2D materials‐based electrodes. Usually, transition metal oxide/chalcogenides and conducting polymers obey the pseudocapacitive charge storage mechanisms. For hybrid supercapacitors, a combination of EDLC and pseudocapacitive materials is considered to achieve high energy density and capacitance values. Hybrid supercapacitors can be classified into asymmetric, composite and battery types depending on their configuration. The asymmetric supercapacitors with different charge storage mechanisms are termed supercapattery, or supercabattery devices, where one electrode is commonly carbon material (EDLC), and the other electrode is battery type or pseudocapacitive material. In recent years, different smart supercapacitors with novel functional components materials and device configurations have been developed which include self‐healing supercapacitors, microsupercapacitor, electrochromic supercapacitors, self‐charged supercapacitors, and integrated supercapacitor devices such as photosupercapacitors, shape memory, photodetection supercapacitor, chemical/biosensor supercapacitor etc.^[^ ^28^, ^29^, ^30^ ^]^

### 2.3 Photosupercapacitors

**Figure 4:**
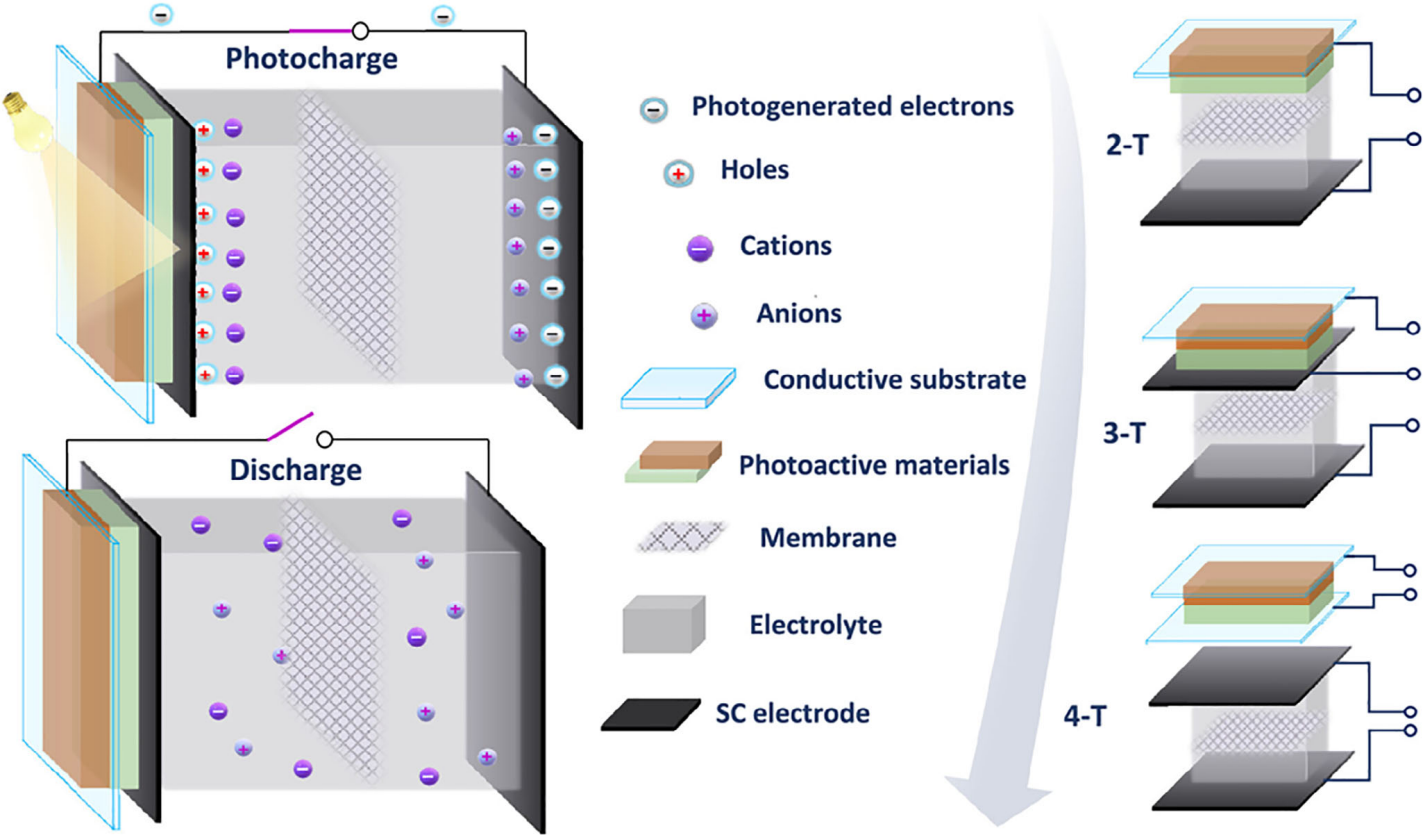
Schematic illustration of the photo charging and dark discharge processes in a photosupercapacitor, and integration of a photosupercapacitor into various terminal configurations: 2‐terminal, 3‐terminal, 4‐terminal. Adapted with permission.^[^ ^31^ ^]^ Copyright 2023, American Chemical Society.

A photosupercapacitor is a single module in which both energy conversion (photovoltaic unit) and a supercapacitor (energy storage unit) are integrated to achieve effective generation and power capability in a single device.^[^ ^19^, ^23^, ^31^ ^]^ Photosupercapacitors can be classified into three, depending on the different terminal configurations used for the integration of photovoltaic and supercapacitors (**Figure** **4**).^[^ ^31^ ^]^ In the first approach, two cells (photovoltaic and supercapacitor) are mechanically connected through an external wire in a 4‐terminal (4T) configuration. In 4T arrangements, the performance of the integrated photosupercapacitor depends on its individual components but it has many problems such as energy losses due to the resistance of the external wire, intricate packaging and lower overall efficiency.^[^ ^32^, ^33^, ^34^ ^]^ In a 3‐terminal (3T) configuration, solar cell and supercapacitor units share one of the electrodes which serves as either a positive or negative electrode, whereas the positive electrode assists a dual function for photo conversion and energy storage in a 2‐terminal (2T) configuration.^[^ ^19^, ^23^, ^31^ ^]^ Though the 2T layout has the advantages of using lower material and substrate, it has drawbacks including low charging voltage, high resistance, low charge‐discharge efficiency, and self‐discharge to the photoactive side.^[^ ^35^, ^36^ ^]^

### 2.4 Key Performance Parameters for Supercapacitors and Photosupercapacitors

The different parameters used for analysis of supercapacitors and photosupercapacitors performance are as follows:^[^ ^3^, ^37^, ^38^ ^]^

#### 2.4.1 Specific Capacitance (C_sp_)

It is the ability of a supercapacitor device to store a charge, which is calculated from cyclic voltammetry (CV) and galvanostatic charge/discharge (GCD) recordings.

(2) $$C_{sp}=\frac{\int IdV}{m.\upsilon .\Delta V}\left(\mathrm{by}\;\mathrm{CV}\right)$$

(3) $$C_{sp}=\frac{I.\Delta t}{m.\Delta V}\left(\mathrm{by}\;\mathrm{GCD}\right)$$

Here, *m* is the mass of active material, υ is the scan rate, ∆*V* is the potential window, and ∆*t* is the discharge time.

#### 2.4.2 Energy Density (ED) and Power Density (PD)

They are used to determine how much energy is stored in the device per unit mass and how quickly it can deliver.

(4) $$ED=\frac{1}{2}C_{sp}{\left(\Delta V\right)}^{2}$$

(5) $$PD=\frac{E}{t}$$

The following formulas were used to assess the supercapacitor devices conversion (η_conv_), storage (η_st_), and overall efficiencies (η_ov_).

(6) $$\eta_{conv}=\frac{E_{conv}}{E_{light}}=\frac{V_{oc}J_{sc}FF}{P_{in}A_{PV}}$$

(7) $$\eta_{st}=\frac{E_{output}}{E_{conv}}=\frac{0.5C_{sp}V^{2}}{P_{in}A_{PV}\eta_{conv}}$$

(8) $$\eta_{ov}=\frac{E_{output}}{E_{light}}=\frac{0.5C_{sp}V^{2}}{P_{in}A_{PV}t_{c}}$$

Here, *V*_oc_ is the open‐circuit voltage, *J*_sc_ is the short‐circuit current density, FF is the fill factor, *P*_in_ is the incident‐light power density, and *A*_PV_ is the surface area of the solar mini‐module.

### 2.5 Mechanism and Integration of Photovoltaic and Supercapacitor Units

The integration of photovoltaic components with supercapacitors into unified, multifunctional systems (photosupercapacitors) represents a transformative strategy for realizing compact, self‐sustaining energy storage technologies. Such integrated architectures not only enable simultaneous solar energy harvesting and charge storage but also significantly reduce device footprint and interfacial energy losses. The underlying integration mechanisms and structural designs are highly dependent on the nature of the photoactive materials and the system configuration. Recent developments span a diverse range of platforms, including organic photovoltaics (OPVs), dye‐sensitized solar cells (DSSCs), photoelectrochemical (PEC) systems, and perovskite‐based hybrids—each offering unique advantages in terms of flexibility, charge transport dynamics, and energy conversion‐storage synergy.

**Figure 5:**
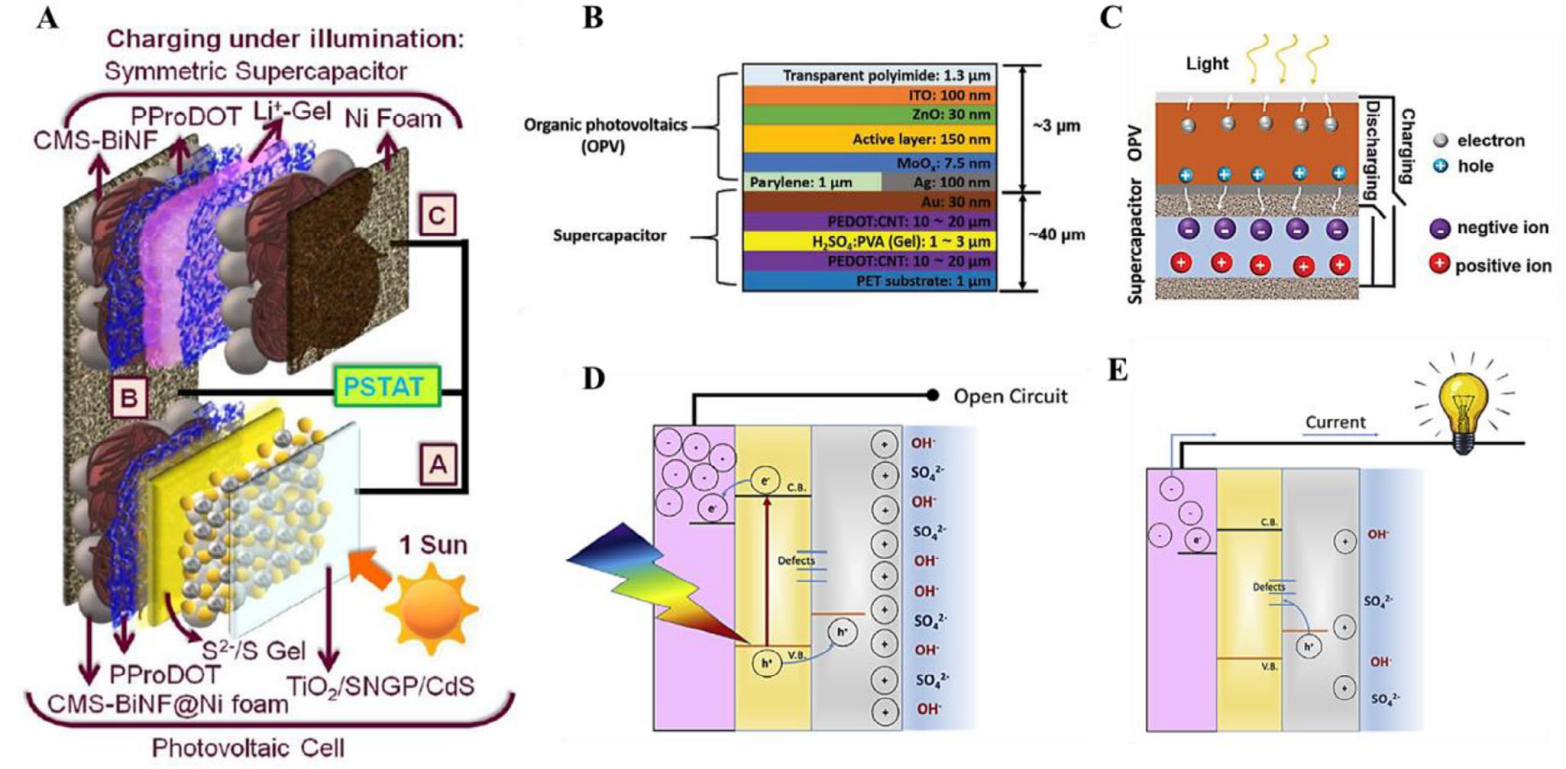
A) Schematic illustration of a photosupercapacitor charging under illumination. Adapted with permission.^[^ ^39^ ^]^ Copyright 2020, Royal Society of Chemistry. B) Schematic illustration of the integrated device fabricated through top‐down method, C) Schematics highlights of the working mechanism of the photocharging process. Adapted with permission.^[^ ^40^ ^]^ Copyrights 2020, Wiley. Schematic depiction of the mechanism of D) charging and E) discharging of BiVO_4_ based photosupercapacitor. Adapted with permission.^[^ ^41^ ^]^ Copyrights 2020, Elsevier.

For instance, the device developed by Das et al. integrates a PV cell and a symmetric SC into a unified system using a shared Ni foam current collector.^[^ ^39^ ^]^ The PV component features a TiO_2_/SNGP/CdS photoanode, where sulfur‐ and nitrogen‐doped graphene particles (SNGP) act as co‐sensitizers alongside CdS quantum dots, broadening light absorption and improving charge separation (**Figure** **5**A). Under illumination, photogenerated electrons from the photoanode are directed to a poly(3,4‐propylenedioxythiophene)/carbon micro‐spheres‐bismuth nanoflakes (PProDOT/CMS–BiNF) composite electrode, which serves dual functions: as the PV cell's counter electrode (reducing polysulfide electrolyte) and as one electrode of the SC. The SC's second electrode is an identical PProDOT/CMS–BiNF layer, forming a symmetric storage unit with a Li⁺‐conducting gel electrolyte. Charge storage occurs via dual mechanisms: (1) Faradaic pseudocapacitance from PProDOT's redox reactions and (2) EDLC, from carbon microspheres (CMS) and bismuth nanoflakes (BiNF). The synergy between these materials enhances conductivity, reduces interfacial resistance, and mitigates polymer degradation during cycling. The system achieves an overall photo‐conversion and storage efficiency of 6.8%, with a 72% energy storage efficiency, outperforming many integrated PV‐SC devices. In another interesting work, Liu et al. have used an ultra‐flexible photo‐charging system integrating OPVs and supercapacitors in a vertically stacked architecture.^[^ ^40^ ^]^ The OPV component features a 3‐µm‐thick structure with a poly[4,8‐bis(5‐(2‐ethylhexyl)thiophen‐2‐yl)benzo[1,2‐b;4,5‐b′]dithiophene‐2,6‐diyl‐alt‐(4‐octyl‐3‐fluorothieno[3,4‐b]thiophene)‐2‐carboxylate‐2‐6‐diyl] with [6,6]‐phenyl‐C71‐butyric acid methyl ester (PBDTTT‐OFT:PC₇₁BM) bulk heterojunction as the photoactive layer, sandwiched between ZnO (electron‐transporting layer) and MoO_x_ (hole‐transporting layer), all fabricated on a 1.3‐µm transparent polyimide substrate (Figure 5B,C). The supercapacitor employs PEDOT:PSS/CNT composite electrodes treated with sulfuric acid to enhance conductivity and porosity, enabling efficient ion storage. During operation, light absorption generates electron‐hole pairs in the OPV, which are separated by the built‐in electric field. The electrons are transferred to one supercapacitor electrode (charging it negatively), while holes migrate to the opposite electrode (charging it positively), driving ion redistribution in the electrolyte until equilibrium is reached at the OPV's open‐circuit voltage. The entire device, with a total thickness below 50 µm, achieves a remarkable 6% total efficiency and retains 94.66% performance after 5000 bending cycles at a 2 mm radius, showcasing its suitability for wearable applications.

Likewise, to work with electrochemical photosupercapacitors, Roy et al. have explored photoelectrochemical supercapacitor (PEC) based on a bilayer electrode composed of bismuth vanadate (BiVO_4_) and reduced graphene oxide (RGO) (Figure 5D,E). In this system, BiVO_4_ acts as the photoactive semiconductor, while RGO serves as the capacitive material.^[^ ^41^ ^]^ Under illumination, BiVO_4_ generates electron‐hole pairs. The photogenerated holes migrate to the BiVO_4_‐electrolyte interface, where they participate in oxidation reactions, while the electrons are transferred to the RGO layer, where they are stored via electric double‐layer capacitance. This direct charge separation and storage mechanism eliminates the need for external wiring or bias. In another work An et al. employed nanoporous Cu@Cu_2_O hybrid electrodes, where Cu_2_O serves as both the photo‐absorber and pseudo‐capacitive material.^[^ ^42^ ^]^ Upon illumination, Cu_2_O absorbs visible light and generates electron‐hole pairs. The holes enhance surface oxidation reactions, increasing available redox sites, while electrons reduce the electrode potential, facilitating charge storage. This light‐induced modulation of charge carrier density promotes deeper proton insertion into the Cu_2_O structure, effectively boosting capacitance. The photocharging mechanism thus leverages photocatalytic enhancement of redox activity, resulting in a substantial increase (≈38%) in charge capacity under illumination compared to dark conditions. In addition to the approaches discussed above, several other strategies have been explored to integrate photovoltaic and supercapacitor components into high‐performance photo‐supercapacitor systems. These include multi‐terminal configurations, shared‐electrode architectures, and tandem‐layered designs employing advanced materials such as quantum dots, MXenes, and transition metal dichalcogenides.^[^ ^43^, ^44^, ^45^, ^46^ ^]^ Such innovations aim to enhance photocharging efficiency, interface compatibility, and mechanical flexibility, thereby broadening the scope for next‐generation, self‐powered energy storage devices.

## 3 Recent Developments on Halide Perovskites for Supercapacitors

### 3.1 Supercapacitors

**Figure 6:**
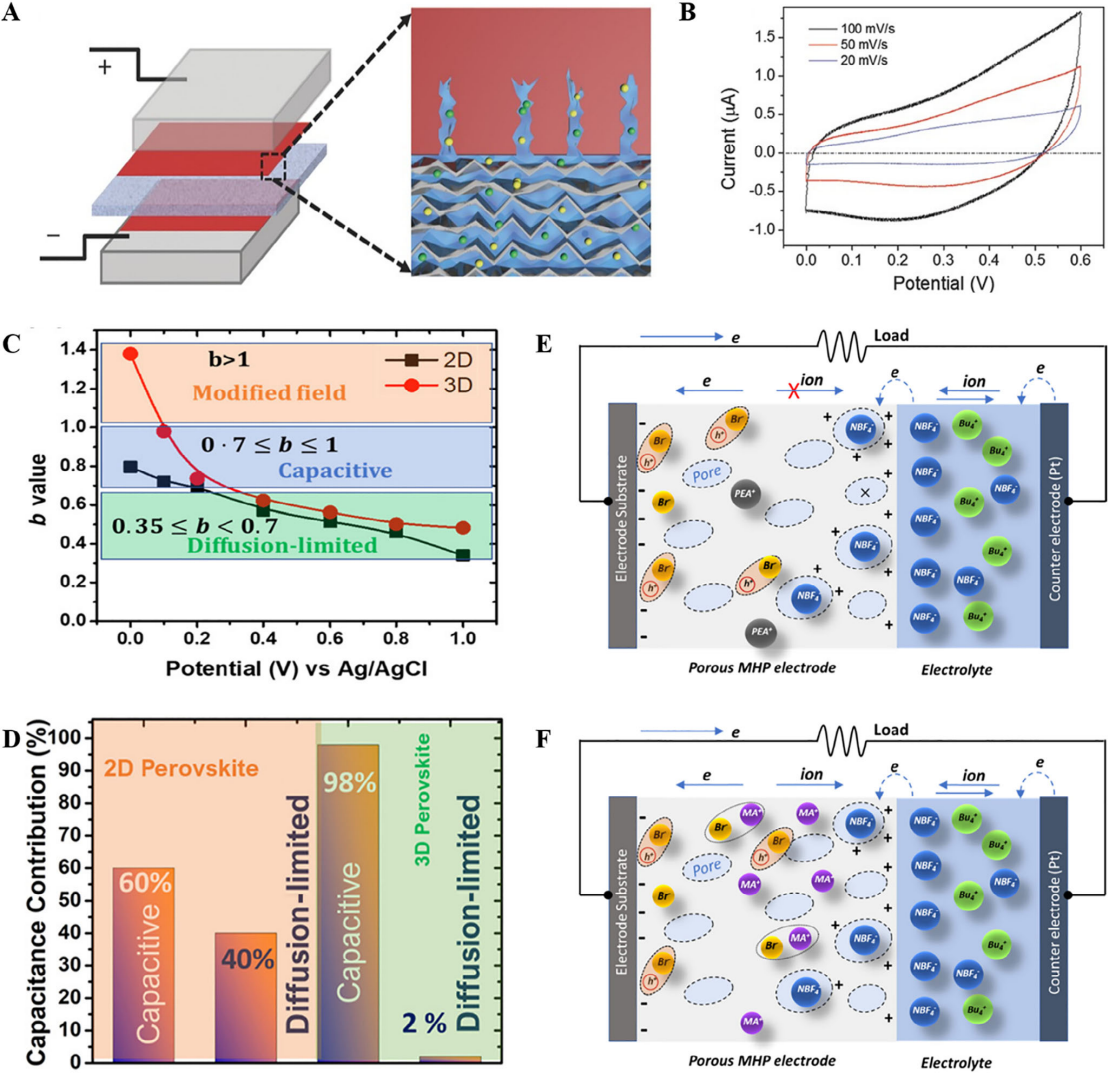
A) Perovskite‐based electrochemical capacitors' cell structure. The interface between the separator's electrolyte and perovskite is zoomed in B) CV profile of MAI/butanol cell. Adapted with permission.^[^ ^50^ ^]^ Copyright 2016, Wiley. C) Variation of the b value as a function of applied potential against Ag/AgCl of perovskite electrodes D) Schematic shows diffusion‐limited and capacitive processes contribution at a low applied field E) The charge storing processes in 2D perovskite‐based supercapacitors F) The charge storing processes in 3D perovskite‐based supercapacitors. Adapted with permission.^[^ ^55^ ^]^ Copyright 2021, American Chemical Society.

Due to their higher ionic conductivity compared to electronic conductivity, along with contributions from their unique crystal structure, reversible active sites, good stability, and abundant oxygen vacancies, halide perovskites are used as active electrode materials for supercapacitor applications.^[^ ^8^, ^10^, ^47^, ^48^, ^49^ ^]^ Zhou et al. first reported a symmetric electrochemical supercapacitor based on MAPbI_3_ perovskite, in which the perovskite not only served as the electrode but also as a solid electrolyte.^[^ ^50^ ^]^ The cell structure of electrochemical capacitors based on perovskite is schematically represented in **Figure** **6**A. A porous ion‐permeable membrane wet in electrolyte separates the two perovskite‐covered flourine‐doped tin oxide (FTO) electrodes. In a half cell of a device, the MAPbI_3_ layer plays a role of the role of a solid electrolyte (FTO/MAPbI_3_ interface) and an electrode (MAPbI_3_/electrolyte interface). The shape of CV curves in Figure 6B indicates that the capacitance is driven primarily by the electric double‐layer(s) that occur from ion adsorption/desorption at the device interface. The fabricated supercapacitor exhibited a capacitance value of 5.8 µF cm^−2^ and 3.68 µF cm^−2^ for perovskite/butanol and 1‐butanol electrolyte‐based devices. MAPbI_3_ dissolution in the electrolyte forms vacancies, which are essential for ion migration in the perovskite. After 10 000 cycles, the capacitance output of these capacitor cells remains steady, demonstrating good cyclability. The flow of free charge carriers in organometal halide perovskites primarily activates the ion production and transport processes, which may also be interface‐dependent. Popoola et al. reported that MAPbI_3_ perovskite‐based symmetric supercapacitor shows 3.65 times greater areal capacitance (21.5 µF cm^−2^) for the quasi‐solid‐state electrolytes as compared to the liquid state electrolytes.^[^ ^51^ ^]^ The device displayed fast discharge properties with the least relaxation time of 251.19 µs, power density of 5.05 W cm^−2^ and cycling stability of 98.34% after 1000 cycles. MAPbBr_3_ single crystal‐based supercapacitor electrodes showed 500 times higher volumetric capacitance (429 F cm^−3^) as compared to the spin‐coated thin films‐based capacitors (0.8 F cm^−3^) due to its high ionic diffusion coefficient (5.61 × 10^−13^ m^2^ s^−1^), lower charge transfer resistance (62.5 Ω cm^−2^) and highly porous isotropic structure with a high degree of micro‐strain.^[^ ^52^ ^]^ The control over lattice defects and crystallite size modulates the ion migration in the materials. By mixing powders of different halide‐based perovskites single crystals, and controlling the particle size by employing a mechanochemical approach, it has been possible to improve the energy storage performance of the fabricated devices by tuning the ionic conductivity of the perovskites.^[^ ^53^, ^54^ ^]^ From a comparative study on 3D bulk and 2D layered halide‐based perovskite supercapacitors, it is observed that the ion kinetics in the active layer contributes to the capacitive mechanism from the pseudocapacitance and EDLC as compared to the diffusion‐controlled mechanism at low applied field.^[^ ^55^ ^]^ Figure 6C shows that the charge storage mechanism in active perovskite electrodes is diffusion‐controlled because of electrolytic ion intercalation and deintercalation. The constraint parameter (*b*) values are in the range of 0.35 < b < 0.7 at higher potentials of >0.2 V. 2D perovskites have a lower ion migration rate than 3D perovskites due to their intercalated spacer ligands and strong van der Waals interactions; thus, the *b* value does not exceed unity in 2D material. At low applied fields, 2D and 3D electrodes contribute 60% and 98% of the total capacitance, respectively, due to large surface polarization and redox reactions (Figure 6D). 3D perovskite‐based supercapacitors showed only 2% diffusion contribution as compared to the 40% contribution for 2D perovskites due to the strong electron‐ion coupling. The schematic in Figure 6E,F illustrates the charge flow and storage process in the 2D layered and 3D bulk perovskite‐based supercapacitors. The field variation caused by ion migration in 2D perovskites is relatively mild, resulting in decreased charge accumulation at the perovskite/electrolyte interface. But, this less ion migration ability makes 2D perovskite devices more stable than 3D perovskite‐based supercapacitors. Rao et al. demonstrated that quasi 2D perovskites obtained by the modification of spacers (Ex: 4‐Fluorobenzylamin hydroiodide spacer cation based) can be an alternative halide with more stable performance as compared to other perovskites.^[^ ^56^ ^]^ The quasi‐2D perovskite with acetylene carbon black displayed a specific capacitance of 3.3 F g^−1^ with an energy density of 170 mWh Kg^−1^, power density of 109 W Kg^−1^ and 91% capacitance retention after 1000 cycles. Similarly, lead‐free perovskites such as MASnCl_3_, MABi_2_I_9_, and other low‐dimensional transition metal (Fe, Co, and Ni) halide perovskite‐based supercapacitors are reported as promising materials for the design of low‐cost electrodes for supercapacitors with high energy density and power density.^[^ ^57^, ^58^, ^59^ ^]^

**Figure 7:**
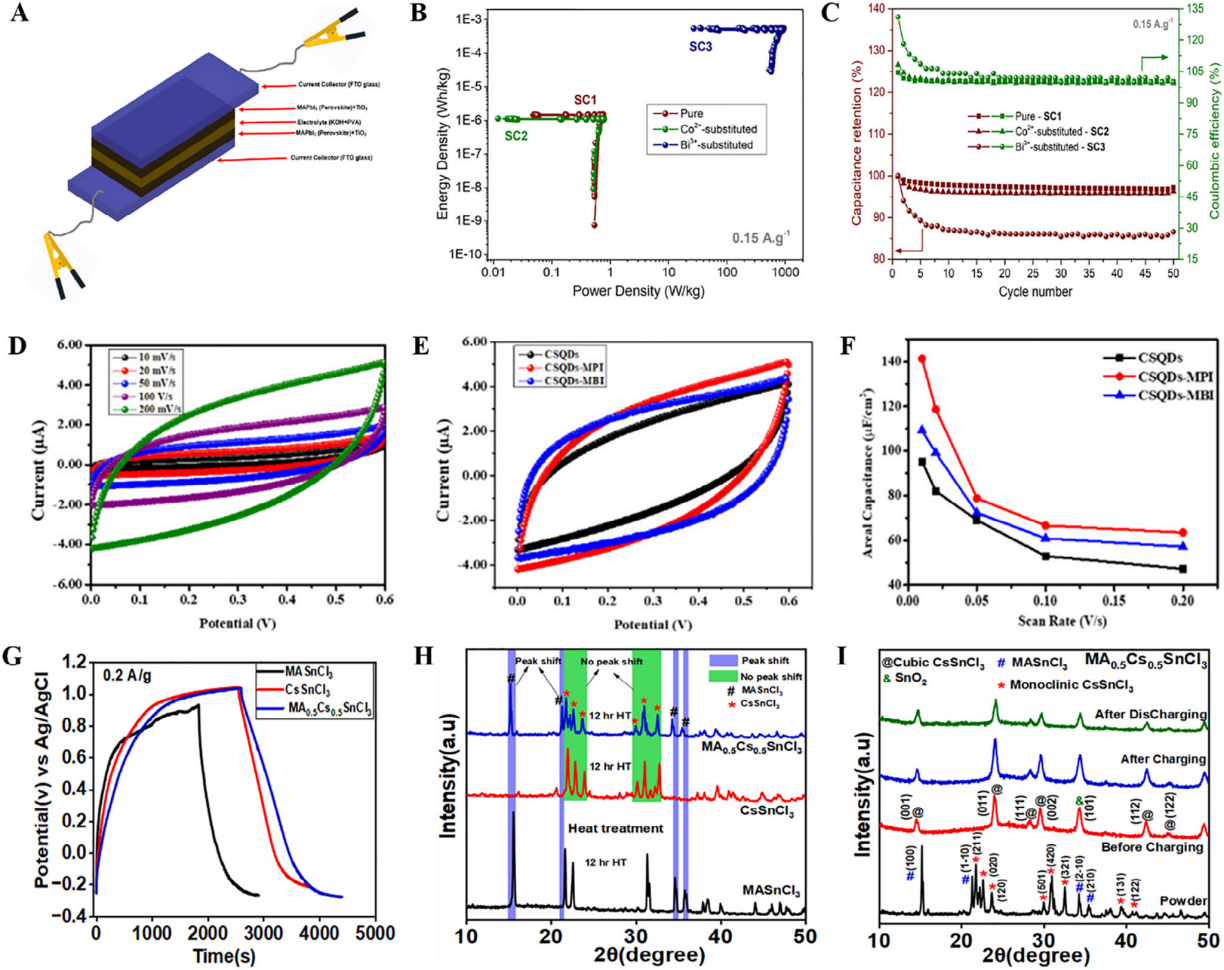
A) Diagrammatic illustration of assembly of perovskite‐based symmetrical supercapacitors B) Ragone plot of supercapacitors C) Capacitance retentions and coulombic efficiencies of supercapacitors under repeated cycling at 0.15 A g^−1^ for 50 cycles. Adapted with permission.^[^ ^61^ ^]^ Copyright 2022, American Chemical Society. D) CV curves of CSQDs‐MPI at different scan rates E) CV curves at 0.1 V s^−1^ scan rates F) Areal capacitances versus scan rates graph. Adapted with permission.^[^ ^64^ ^]^ Copyright 2020, Wiley. G) GCD curves at 0.2 A g^−1^ H) XRD pattern of perovskite powders at 120 °C for 12 h I) MA_0.5_Cs_0.5_SnCl_3_ electrode before and after the charging/discharging process. Adapted with permission.^[^ ^38^ ^]^ Copyright 2025, Elsevier.

By doping different metal ions, the properties of the halide perovskites can be tailored since it is related to the Goldschmidt tolerance factor, which leads to achieving improved optoelectronic and electrical properties, surface area, and tunable pore size.^[^ ^60^, ^61^ ^]^ The 2% Cu‐doped CH_3_NH_3_PbI_3_ perovskite thin film exhibits a specific capacitance of 761 F g^−1^ at a 10 mV s^−1^ scan rate.^[^ ^60^ ^]^ At higher concentrations, the structure loses its homogeneity and copper precipitation forms. Also, an increase in the concentration of copper in the structure will cause a resistance between the bands, which decreases the load storage capacity. In a similar work, Güz et al. have demonstrated that the substitution of Pb^+2^ ions in MAPbI_3_ with Bi^3+^ and Co^2+^ ions significantly alters its electrochemical and electronic properties.^[^ ^61^ **^]^** **Figure** **7**A shows a schematic figure of the assembly of symmetric supercapacitors. Bi^3+^ substituted MAPbI_3_ perovskite‐based supercapacitors exhibited a much‐improved power density of 934.6 W Kg^−1^ due to their favorable effect on the rapid migration of large ions at the electrode and electrolyte interface (Figure 7B). The supercapacitor devices made of Co^2+^‐substituted and Bi^3+^‐substituted perovskite electrodes retained 96.3% and 86.6% of their initial capacitances after 50 cycles of charging and discharging. Their Coulombic efficiencies are 99.5% and 99.8%, respectively (Figure 7C). Since the theoretically predicted quantum capacitance is the quotient of the differential of charge density and local potential, the supercapacitor performance of an electrode material can be further improved by increasing the number of states near the Fermi level by appropriate modification and by making a hybrid material.^[^ ^62^, ^63^ ^]^ By keeping this in mind, Oloore et al. designed supercapacitor electrodes based on CdS quantum dots‐MAPbI_3_, which exhibited an areal capacitance of 141 µF cm^−2^, an energy density of 23.8 nWh cm^−2^, and stability of 87% up to 4000 cycles (Figure 7D–F).^[^ ^64^ ^]^ This enhanced performance results from the perovskite material's ability to donate more charges and enhance ionic conductivity through its crystals. Moreover, organometallic halide perovskites can reduce the paths for the transport of charges or ions by offering more active sites for electrochemical processes. Similarly, carbon nanodots anchored on lead and bismuth‐based perovskites facilitated enhanced electron transfer and cation accessibility to faradaic active sites, leading to better energy storage performance.^[^ ^65^ ^]^ In the MA_0.5_Cs_0.5_SnCl_3_ perovskites, A‐site occupation by MA^+^ and Cs^+^ cations creates a disordered arrangement that results in nanoscale phase segregation.^[^ ^38^ ^]^ This helps to improve the supercapacitor's performance and its stability. The GCD curves in Figure 7G show that MA_0.5_Cs_0.5_SnCl_3_ perovskites have a high time of charging and discharging compared to MASnCl_3_ and CsSnCl_3_. The energy density over 60 Wh kg^−1^ was shown by MA_0.5_Cs_0.5_SnCl_3_, which was 1.6 times higher than that of the supercapacitor based on MASnCl_3_. The X‐ray diffraction (XRD) peak position of MA_0.5_Cs_0.5_SnCl_3_ perovskite shifted toward a lower angle due to the presence of a microstrain, as shown in Figure 7H. This strain expands the lattice at the MA site takes place. The MASnCl_3_ phase is transformed into amorphous SnO_2_, whereas the monoclinic CsSnCl_3_ phase is transformed into the cubic phase during electrode preparation. The crystal structure of MA_0.5_Cs_0.5_SnCl_3_ changes to the cubic phase of CsSnCl_3_ in the charging and discharging process, which improves stability (Figure 7I). The MA_0.5_Cs_0.5_SnCl_3_ perovskite electrode retained around 99.80% of its initial capacitance after 2000 cycles. This is higher compared to the CsSnCl_3_ electrode (98%) and the MASnCl_3_ electrode (76%).

**Figure 8:**
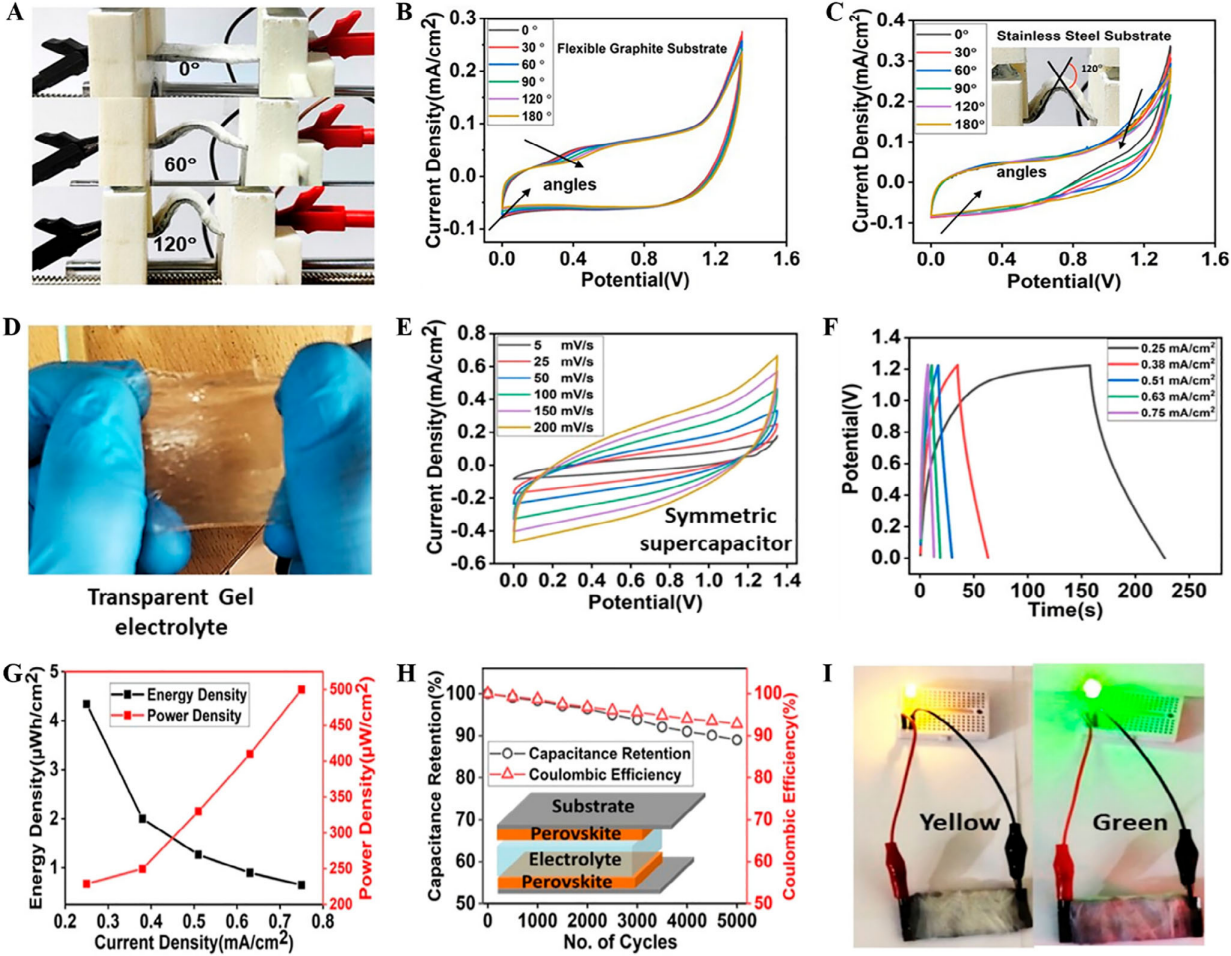
A) Flexible supercapacitor bending images B) CV curves recorded for a flexible graphite substrate at different bending angles C) CV curves recorded for a stainless steel substrate at different bending angles. Adapted with permission.^[^ ^72^ ^]^ Copyright 2023, American Chemical Society. D) Transparent gel electrolyte with Li‐ion (E) CV curves recorded for symmetric device F) GCD curves recorded for the symmetric device G) Energy and power density plots H) Stability tested over 5000 cycles I) Supercapacitor devices supporting different colored LEDs. Adapted with permission.^[^ ^73^ ^]^ Copyright 2023, American Chemical Society.

**Figure 9:**
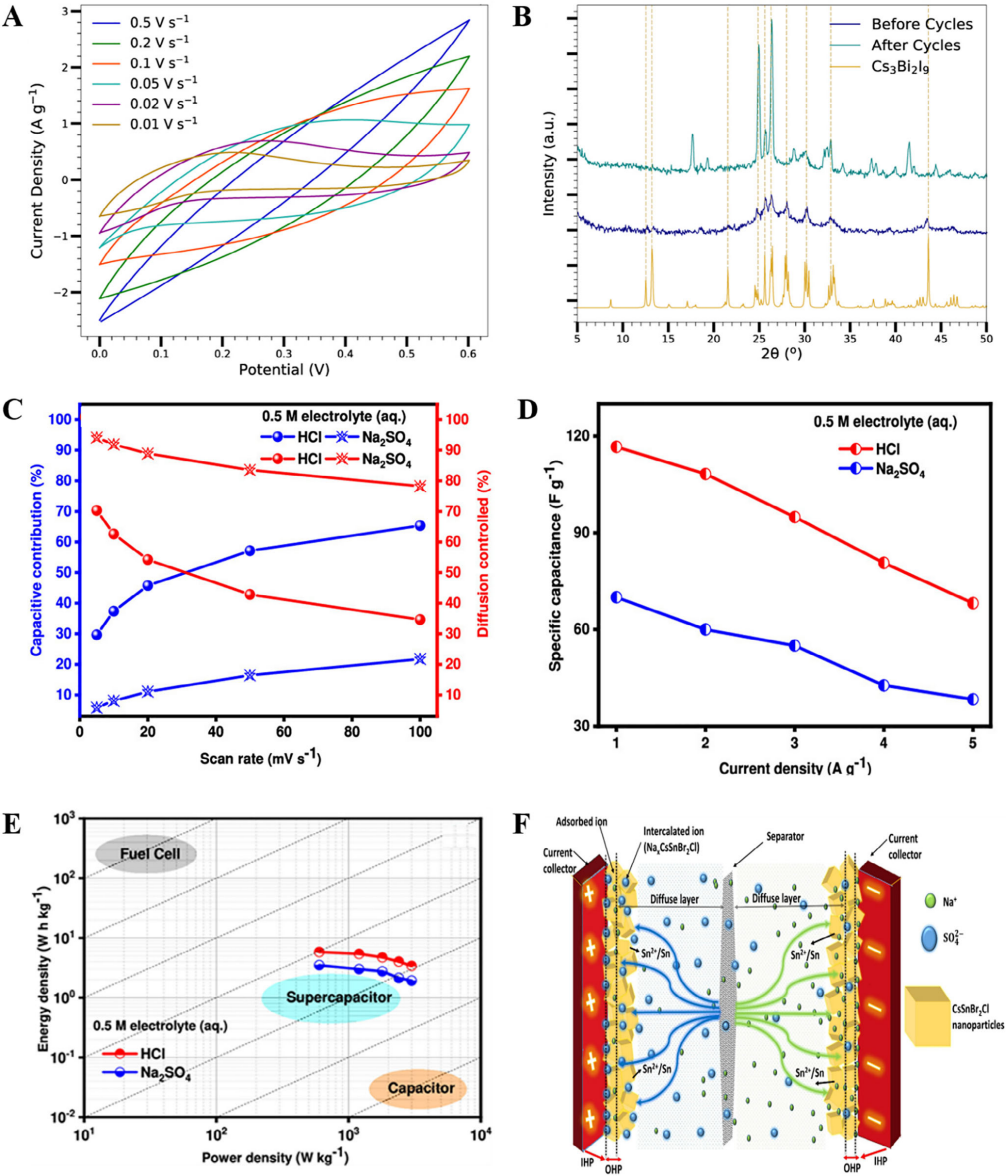
A) Cyclic voltammograms of the supercapacitor at different scanning rates B) XRD patterns of the electrode surface before and after 5000 charge/discharge cycles at 1.0 A g^−1^. Adapted with permission.^[^ ^76^ ^]^ Copyright {2019}. Institute of Physics. C) contribution ratio of the capacitive and diffusion charge versus scan rates D) variation of specific capacitance with current densities E) Ragone plots of CsSnBr_2_Cl electrode material in the same electrolyte solutions F) Schematic representation of the charge storage mechanism of the electrode material for both anode and cathode and aqueous 0.5 M Na_2_SO_4_ solution. Adapted with permission.^[^ ^77^ ^]^ Copyright 2024, American Chemical Society.

Cesium‐based perovskites are considered to possess relatively higher stability and have benefits due to their higher absorptivity coefficient, excellent defect tolerance factors, and tunable band gap as compared to the organic cations‐based perovskite materials.^[^ ^66^, ^67^, ^68^, ^69^, ^70^, ^71^ ^]^ Yadav et al. reported CsPbBr_3_ perovskite‐based supercapacitors with lithium bis(trifluromethanesulfonyl)imide (LiTFSi) as the electrolyte to achieve improved energy and power density.^[^ ^72^, ^73^ ^]^ Asymmetric supercapacitor fabricated on graphite substrate and activated carbon as the other electrode showed areal energy density ∼ 8.25 µWh cm^−2^ and good flexibility and stability with bending/twisting ability.^[^ ^72^ ^]^ **Figure** **8**A shows images of flexible supercapacitors at different bending angles between 0 and 180°. The CV curve for the flexible graphite substrate shows a small variation, but the stainless‐steel substrate shows notable alterations, as shown in Figure 8B,C. Also, the device can be utilised with both 0° and 50° bending directions without compromising performance. After 200 bending cycles between 30° and 60°, the stainless‐steel substrate's capacity retention is less than 80%. The supercapacitor's charge‐transfer resistance rises as the bending angle increases for stainless steel substrates, whereas it stays nearly constant for flexible graphite substrates. Figure 8D shows an image of a quasi‐solid‐state gel electrolyte containing Li‐ion used to fabricate CsPbBr_3_ symmetric supercapacitors.^[^ ^73^ ^]^ It is more convenient to use solid‐state supercapacitors with flexible electronics than pouch cells. Specific capacitance of 17 mF cm^−2^ with energy density ≈4.58 µW cm^−2^, stability of ≈89% (5000 cycles) and 91% coulombic efficiency were achieved for a symmetric thin‐film supercapacitor based on CsPbBr_3_ perovskites (Figure 8E–H). Figure 8I illustrates that strong green LEDs (>2.0 V) can be supported by a single device. Ex‐situ spectroscopic studies revealed that the reversible Li‐ion intercalation/deintercalation process contributed to the charge storage performance, leading to improved energy density. Lead‐free halide perovskite, Cs_3_Bi_2_Cl_9_ showed 8–10 times higher capacitance, 3–4 times higher energy density as compared to the lead‐based perovskites such as MAPbCl_3_ and CsPbCl_3_.^[^ ^74^ ^]^ Three‐electrode‐based CsPb_2_Br_5_ perovskite supercapacitors displayed promising energy storage performance with a specific capacitance of 886 F g^−1^, 76% stability, and 99% coulombic efficiency after 5000 cycles.^[^ ^75^ ^]^ Cs_3_Bi_2_I_9_‐based symmetric supercapacitor with NaClO_4_ as the electrolyte delivered promising capacitance values (areal capacitance ≈2.4 F cm^−2^, specific capacitance ≈280 F g^−1^) and stability ≈88% after 5000 cycles [**Figure** **9**A].^[^ ^76^ ^]^ At high scan rates, the electrolytic Na^+^ and ClO^4−^ ions have less time to form an electrostatic double layer at Cs_3_Bi_2_I_9_ electrodes. At intermediate scan rates, EDLC‐type curves were formed, which indicates that charge storage happened between the coated electrodes and the polarized electrolyte. The low scan rates give the electrolytic ions time to penetrate the activated Cs_3_Bi_2_I_9_ layer and adsorb/desorb Faradaically to the surfaces and near‐surface pockets of the electrodes. Figure 9B shows that the Cs_3_Bi_2_I_9_ active layer maintains its crystal shape over 5000 cycles with minimal chemical degradation, even under extreme electrical conditions. The electrode shows 88% capacitance retention after 5000 cycles. Yasmeen et al. investigated the comparative energy storage performance of CsSnBr_3_ and Cl‐incorporated CsSnBr_2_Cl nanoparticles in different electrolytes (acidic HCl, neutral Na_2_SO_4_, alkaline KOH).^[^ ^77^ ^]^ The CsSnBr_2_Cl perovskite displayed impressive capacitance values of ≈474 F g^−1^ in HCl electrolyte, high operating voltage up to 1.6 V in Na_2_SO_4_, and effective water‐splitting electrodes in KOH [Figure 9C–E]. Supercapacitors based on the CsSnBr_2_Cl performed stable operations in the temperature range of 3–60 °C, and showed an energy density ≈5.83 Wh Kg^−1^ at a power density of 600 W Kg^−1^. Figure 9F shows the schematic representation of the charge storage mechanism of the symmetric supercapacitor devices in Na_2_SO_4_ electrolyte, to which both EDLC and diffusion‐controlled mechanisms contributed.

**Figure 10:**
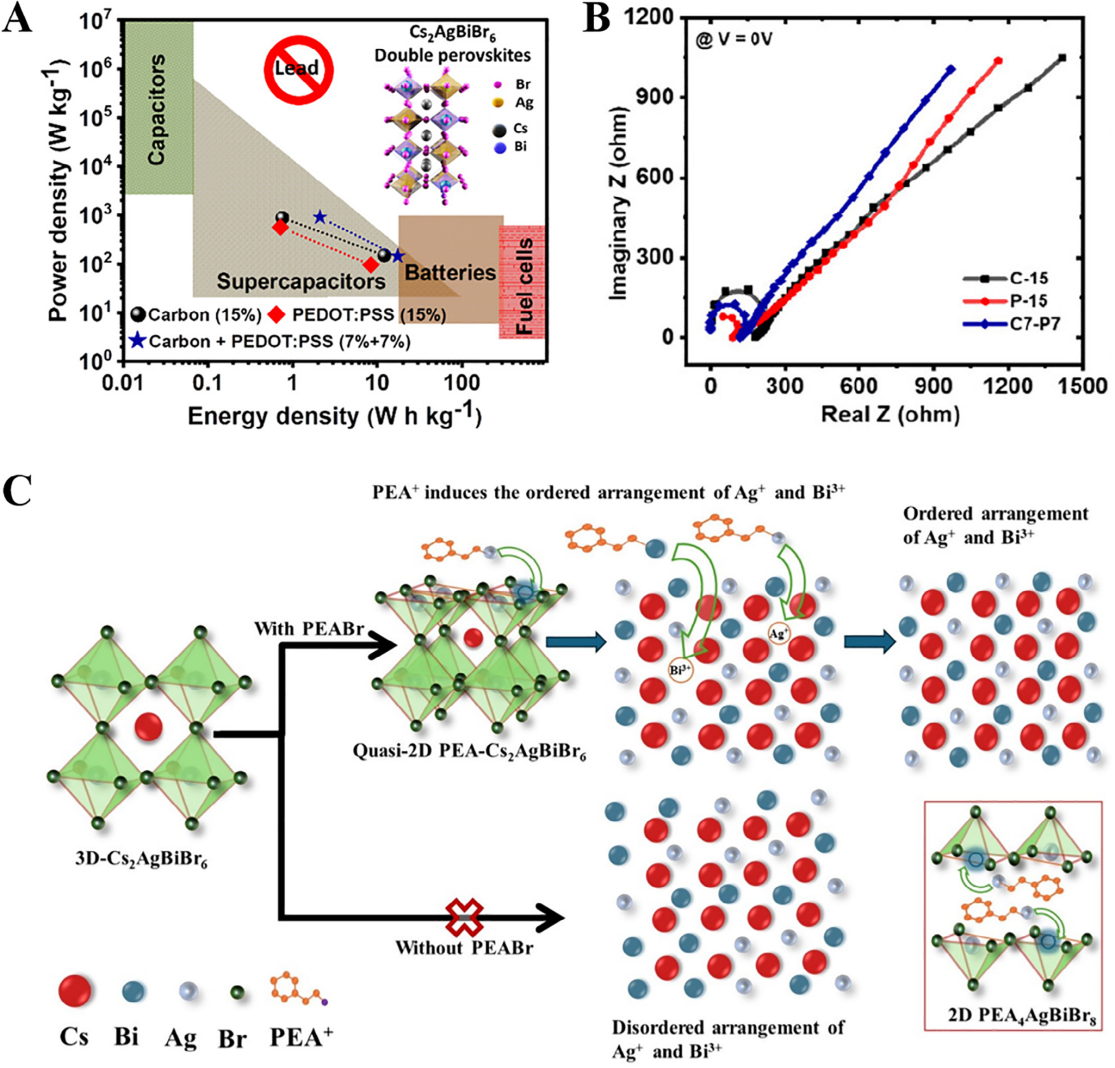
A) Cs_2_AgBiBr_6_ Double Perovskite Ternary Composite with PEDOT:PSS and Carbon Black for Supercapacitor Application B) Nyquist's graphs. Adapted with permission.^[^ ^78^ ^]^ Copyright 2023, American Chemical Society. C) Tuning the energy storage performance and stability of lead‐free quasi‐2D halide perovskite supercapacitor through Ag^+^/Bi^3+^ cation interaction. Adapted with permission.^[^ ^79^ ^]^ Copyright 2024, Elsevier.

Composites of Cs_2_AgBiBr_6_ incorporated with electronic and ionic conductive agents such as carbon black and polymer poly(2,3‐dihydrothieno‐1,4‐dioxin)‐poly (styrene sulfonate) (PEDOT:PSS) conducting polymer improved the specific capacitance and energy density by over 40%, as shown in **Figure** **10**A.^[^ ^78^ ^]^ Optimal performance in lead‐free perovskite‐based supercapacitors depends on balanced electrical and ionic conductivities, as revealed by electrochemical impedance spectroscopy analysis (Figure 10B). The symmetric solid‐state supercapacitor device fabricated with these materials maintains its cyclic voltammetry characteristics also at a high scan rate of 160 mV s^−1^ and exhibits stable performance over a thousand cycles. Yadav et al. demonstrated that bulky organic ammonium cations (PEA^+^) induced the higher ordering arrangements of Ag^+^ and Bi^3+^ in Cs_2_AgBiBr_6_ with improved crystallinity and reduced defect sites (Figure 10C).^[^ ^79^ ^]^ This quasi‐2D perovskite is reported to be more efficient and stable compared to the pure 3D and 2D perovskites and showed 4.5‐ and 1.75 times higher energy density, respectively. Ag^3+^ ions promote stable complex formation, which increases the overall stability of 2D and quasi‐2D perovskites. Ion migration may have a major impact on the electrochemical supercapacitor's stability and performance. After 2000 cycles, the quasi‐2D halide perovskite electrode device remains capacitive to the extent of 85%. Lead‐free Bi‐based perovskites (Cs_3_Bi_2_Br_9_ and Cs_3_Bi_2_Cl_9_) are found to be much improved stable due to their well‐defined cubic phase and high dielectric constant of bismuth, allowing effective screening of charged defects within the substance.^[^ ^79^, ^80^ ^]^ Due to these reasons, lead‐free vacancy‐ordered A_3_B_2_X_9_ halide perovskite‐based supercapacitors are reported to show better energy storage performance in terms of long cycling stability and energy density. The introduction of oxygen vacancies or defects during the fast‐cooling synthesis of Cs_3_Bi_2_Br_9_ improves its capacitance from 30 to 35.2 F g^−1^.^[^ ^80^ ^]^ The dielectric layer formed through the oxidation process during synthesis produced a high energy density and high porosity, which assists in fast charge and discharge. The fast‐cooled Cs_3_Bi_2_Br_9_ exhibits a higher concentration of oxygen vacancies and active sites than the slowly cooled Cs_3_Bi_2_Br_9_ sample. Similarly, Cs_2_SnX_6_ lead‐free perovskite possesses good stability and less toxicity, and the Sn atom is in a +4‐oxidation state, which is an ideal candidate for the Pb replacement. Vacancy‐ordered Cs_2_SnI_6_‐based solid‐state supercapacitors attained an areal capacitance of 10 mF cm^−2^, energy density of 3.2 µW cm^−2^ at 0.2 mA cm^−2^, and 670 µW cm^−2^ at 0.75 mA cm^−2^, and a high working window of 1.8 V.^[^ ^81^ ^]^ A flexible symmetric device fabricated using this perovskite structure shows high capacitance retention of around 94% up to 180° angles and 93% retention of capacitance after 200 cycles. In this vacancy‐ordered double perovskites like Cs_2_SnI_6_, the absence of A‐site cations and the presence of Sn⁴⁺ coordination vacancies contribute to a well‐defined open framework that supports high ion mobility, broad voltage windows, and mechanical flexibility. Some of the other inorganic lead‐free halide perovskites such as LiZrBr_3_, RbGeI_3_, KCdCl_3_, KNiF_3_, KCuCl_3_, KNiBr_3_, KMnCl_3_, and their composites with rGO, activated carbon, C_60_, and polyaniline, displayed promising performance as the electrode materials for supercapacitors.^[^ ^78^, ^79^, ^80^, ^81^, ^82^, ^83^, ^84^, ^85^, ^86^, ^87^, ^88^ ^]^ **Table** **1** summarizes the performance of different halide perovskite‐based supercapacitors. Vacancy defects, particularly halide and oxygen vacancies, are now recognized as critical features influencing the electrochemical performance and stability of halide perovskites in supercapacitor applications. These intrinsic or engineered defects play a dual role, that is, they not only enhance ionic conductivity by providing low‐energy migration pathways for ion transport but also contribute to the formation of reversible redox‐active sites that support pseudocapacitive behavior. These examples underscore that defect engineering, that is, through vacancy creation, anion/cation substitution, or controlled crystallization, offers a powerful lever to tune ionic pathways, increase charge storage density, and stabilize electrode interfaces.

**Table 1:** Performance of supercapacitors based on halide perovskites.

Halide perovskite	Types of supercapacitors	Electrolyte	Performance	Stability [%; cycles]	Refs.
MAPbI_3_	Areal, symmetric	KOH+PVA	C_sp_ = 21.5 µF cm^−2^; PD = 5.05 W cm^−2^	98.3%; 1000	[51]
MAPbBr_3_	Volumetric, symmetric	LiTFSi	C_sp_ = 429 F cm^−3^; ED = 12.75 Wh Kg^−1^; PD = 225 W Kg^−1^	97%; 1500	[52]
MAPbI_3_ + MAPbBr_3_	Gravimetric, areal, symmetric	LiTFSi	C_sp, grav_ = 70 F g^−1^; C_sp, areal_ = 88.25 mF cm^−2^; ED = 22.75 Wh Kg^−1^; PD = 600 W Kg^−1^	97%; 1000	[53]
MAPbBr_3_	Volumetric, areal, symmetric	LiTFSi	C_sp_ = 36.8 F g^−1^; C_sp, areal_ = 58 mF cm^−2^; ED = 9 Wh Kg^−1^ PD = 400 W Kg^−1^	98%; 1000	[55]
MABi_2_I_9_	Areal, symmetric	LiTFSi	C_sp_ = 5.5 mF g^−1^	85%; 10 000	[57]
Bi and Co doped MAPbI_3_	Areal, symmetric	KOH+PVA	C_sp_ = 2.13 µF cm^−2^; PD = 934.6 W Kg^−1^	86%; 56	[61]
CdS‐ MAPbI_3_	Areal, symmetric	KOH+PVA	PD = 12.7 mW cm^−2^	87%; 4000	[64]
C dot‐ MAPbI_3_	Gravimetric, symmetric	H_3_PO_4_+PVA	C_sp_ = 402 F g^−1^; ED = 55.8 Wh Kg^−1^;	99%; 2800	[65]
CsPbBr_3_	Areal, asymmetric	LiTFSi	ED = 8.25 µWh cm^−2^ PD = 640 µW cm^−2^	–	[72]
CsPbBr_3_	Areal, symmetric	LiTFSi	C_sp_ = 17 mF cm^−2^; ED = 4.58 µWh cm^−2^; Coulombic efficiency = 91%	89%; 5000	[73]
Cs_3_Bi_2_Cl_9_	Areal, symmetric	LiTFSi	C_sp_ = 64 mF cm^−2^; ED = 6.5 µWh cm^−2^	90%; 1500	[74]
CsPb_2_Br_5_	Gravimetric, symmetric	Na_2_SO_4_	C_sp_ = 886 F g^−1^; Coulombic efficiency = 99%	76%; 5000	[75]
Cs_3_Bi_2_I_9_	Areal, Gravimetric	NaClO_4_	C_sp, grav_ = 280 F g^−1^; C_sp, areal_ = 2.4 F cm^−2^	88%; 5000	[76]
CsSnBr_2_Cl	Gravimetric, symmetric	HCl, Na_2_SO_4_, KCl	In (HCl): C_sp_ = 474 F g^−1^; ED = 5.83 Wh Kg^−1^; PD = 600 W Kg^−1^; Coulombic efficiency = 91%	93%; 5000	[77]
Cs_2_SnI_6_	Areal, symmetric	LiTFSi	C_sp_ = 10 mF cm^−2^; ED = 3.2 µWh cm^−2^; PD = 670 µW cm^−2^; Working window = 1.8V	–	[81]

### 3.2 Photosupercapacitors

**Figure 11:**
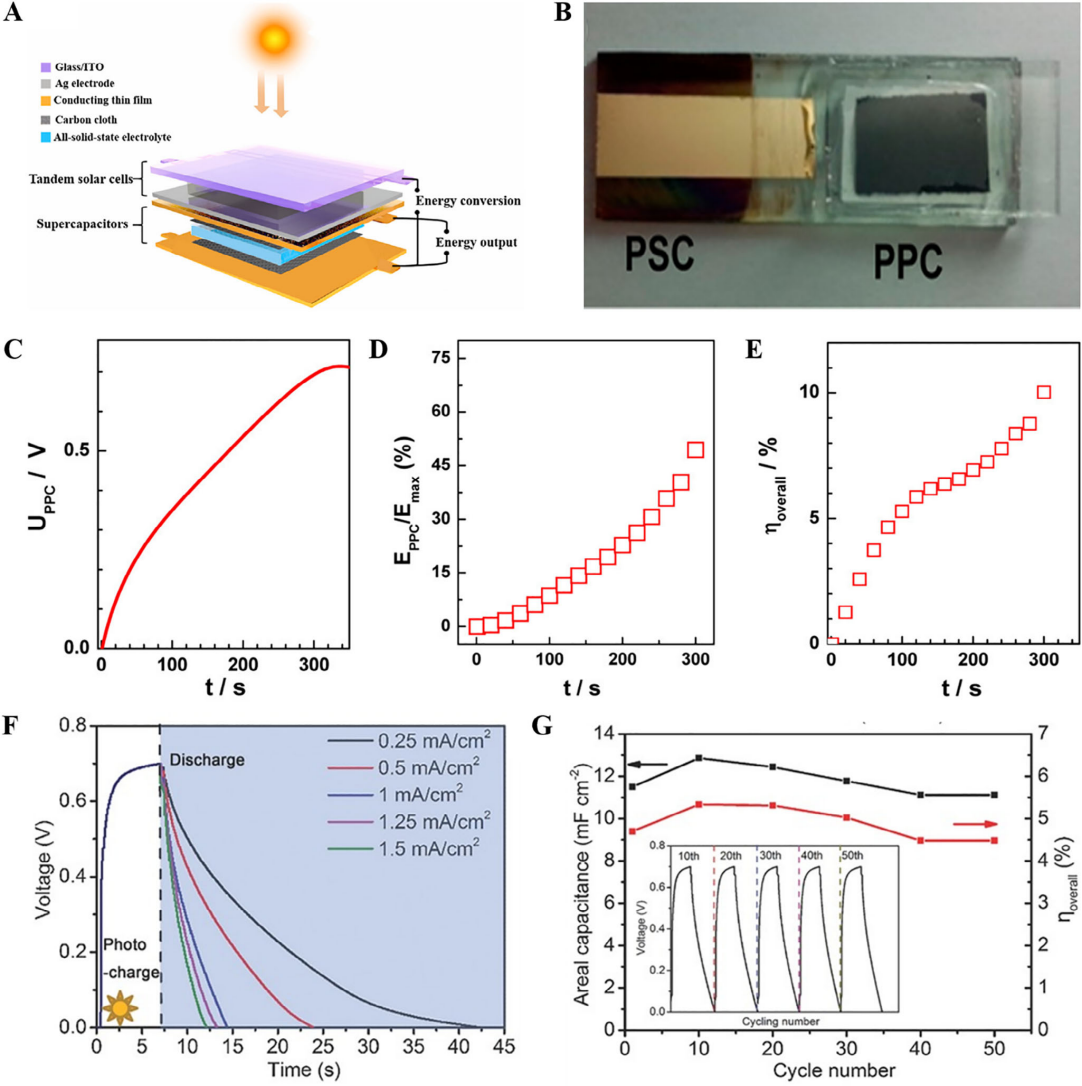
A) Schematic representation of tandem solar cells integrated with the solid‐state asymmetric supercapacitors. Adapted with permission.^[^ ^91^ ^]^ Copyright 2020, Elsevier. B) photograph of the device C) Charging curve with an illuminated area of 0.06 cm^2^ as the power source D) the energy storage proportion E) overall energy conversion of the stored solar energy versus the solar‐charging time. Adapted with permission.^[^ ^99^ ^]^ Copyright 2015, American Chemical Society. F) The voltage transients of the photosupercapacitor device during photocharge under AM 1.5 simulated sunlight G) Photo‐charge/galvanostatic discharge cycling stability. Adapted with permission.^[^ ^100^ ^]^ Copyright 2016, Wiley.

**Figure 12:**
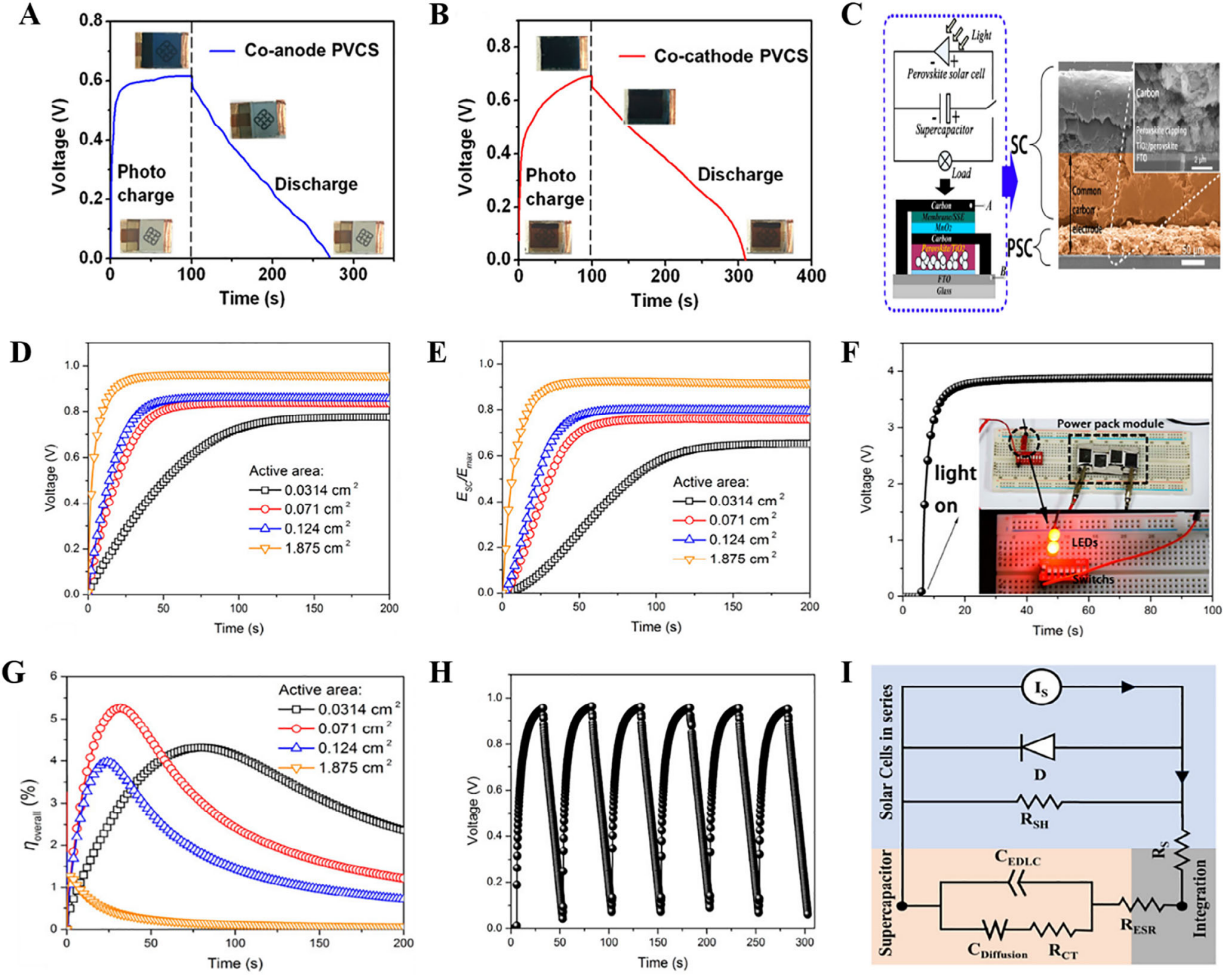
A‐B) The co‐anode and co‐cathode device's voltage‐time curves for the photocharging process within 100 s and the discharging process at 0.1 mA cm^−2^ current density. Adapted with permission.^[^ ^101^ ^]^ Copyright 2016, American Chemical Society. C) Schematic and cross‐sectional SEM image of the integrated device D) Photocharging curves of the power pack E) energy storage proportion (χ) of the supercapacitor F) overall energy conversion against the solar‐charging time G) photocharging/galvanostatic discharging curve of the power pack H) Solar‐charging curve of a tandem system. The photograph of the power pack module and LEDs. Adapted with permission.^[^ ^102^ ^]^ Copyright 2017, American Chemical Society. I) Equivalent circuit of integrated solar cell and supercapacitor. Adapted with permission.^[^ ^104^ ^]^ Copyright 2024, American Chemical Society.

Since the first conceptual research on “photosupercapacitor” by Tsutomu Miyaska in 2004, there has been growing interest in integrating different solar cells and supercapacitors.^[^ ^3^, ^89^ ^]^ These systems are useful in self‐powered gadgets, which need direct conversion and storage of solar power. However, the integration of the two devices (solar cells and supercapacitors) has several challenges to achieving reproducibility, reliability, and long cycling performance. In this area, halide perovskites have attracted a lot of interest because of their tunable bandgap, high carrier mobility, high optical absorption, and ambipolar charge transport properties.^[^ ^90^ ^]^ Though their optoelectronic qualities are intriguing for the photoconversion of light, they have no direct bearing on capacitance or energy storage behavior. The organic halide‐based perovskites have shown great potential for the advancement of high‐performance photosupercapacitors.^[^ ^10^, ^19^, ^25^, ^91^, ^92^, ^93^, ^94^, ^95^, ^96^, ^97^ ^]^ The combination of halide perovskites with other materials (like carbon) helps to create efficient energy conversion‐storage interfaces.^[^ ^98^, ^99^, ^100^ ^]^ This interface allows better light conversion and also generates higher electrochemical activity. In 2015, Du et al. first reported the integration of MAPbI_3‐x_Cl_x_ perovskite‐based solar cells with a flexible solid‐state supercapacitor based on self‐stacked solvated graphene.^[^ ^98^ ^]^ The integrated photosupercapacitors can charge the supercapacitor under AM 1.5 illumination but discharge at 0.75 V, a voltage lower than the open‐circuit voltage (V_OC_) of the solar cell (0.9 V); with 45 s discharge time. The obtained lower discharge voltage is attributed to the connection loss by the external wire and from the photocharging process of the integrated device. **Figure** **11**A shows the schematic illustration of the device structure of the wireless portable lightweight self‐charging power packs by tandem solar cells integrated with the solid‐state asymmetric supercapacitors.^[^ ^91^ ^]^ The device displays a specific capacitance of 234 mF cm^−2^ at 1 mA cm^−2^ current density and 10 mA cm^−2^ current density, which decreases to 100 mF cm^−2^. It shows an overall efficiency of 12.43% at 1 mA cm^−2^ and energy storage efficiency of 72.4%. The study showcased the development of wireless portable lightweight self‐charging power packs using tandem solar cells and solid‐state asymmetric supercapacitors as energy storage devices is advantageous. Kumar et al. showed that the mixed halide perovskites (CH_3_NH_3_PbBr_2_I)‐based electrode exhibited photocapacitance improvement up to 15 F g^−1^, whereas photocapacitance decreased up to 12 F g^−1^ by the electrode made from a blended mixture of CH_3_NH_3_PbBr_3_ and CH_3_NH_3_PbI_3_.^[^ ^94^ ^]^ The reason behind performance declines in blend perovskites is photogenerated trapping at nanoscale phase segregation. Also, in comparison to mixed halide perovskite electrodes, these electrodes degrade more because of increased iodine outflow. The mixed halide perovskite electrode shows a photoenergy density of 0.655 Wh kg^−1^ and 1.6 Wh kg^−1^ under low light illumination (1500 LUX) and blue light illumination (90 mW cm^−2^, 405 nm), respectively. A mixed halide perovskite electrode exhibits lower ion migration than a blended perovskite electrode because the halide ions are uniformly mixed in perovskite nanocrystals, thereby preventing phase segregation. Xu et al. reported a photosupercapacitor device with a MAPbI_3_‐based solar cell connected in series along with polypyrrole‐based supercapacitors (Figure 11B).^[^ ^99^ ^]^ If the supercapacitor is connected in series with the solar cell, the stored energy can be released in the dark or used to increase the solar cell's output power. As shown in Figure 11C, a sudden rise is observed in the capacitor voltage to 0.3 V and then slowly reaches a value of 0.710 V. During the solar charging process, the energy storage proportion (φ) reaches nearly 49% with a long charging time of 300 s. The photosupercapacitor device achieved an energy storage efficiency of 10% with an output voltage of 1.45 V under AM 1.5G illumination (Figure 11D,E). Integrated photosupercapacitor based on MAPbI_3_ as solar cell component and PEDOT‐carbon electrode as the supercapacitor component exhibited maximum overall efficiency ≈4.70%, with high energy storage efficiency of 73.77%.^[^ ^100^ ^]^ The capacitor was charged to 0.70 V in 7 s under visible light irradiation, which is close to the perovskite solar cell's as‐prepared open‐circuit value of 0.71 V (Figure 11F). After that, it discharged in the dark at current densities ranging from 0.25 to 1.5 mA cm^−2^. The range of the measured areal capacitance is 12.8 to 10.8 mF cm^−2^. Their photocharge and galvanostatic discharge curves show good stability and slight deviation at the 50th cycle (Figure 11G). The charge and discharge times at this cycle are 7.1 and 7.41 s, respectively. Zhou et al. reported photovoltachromic supercapacitors (PVCCs) by integrating semitransparent MAPbI_3‐x_Cl_x_ perovskite‐based solar cells and electrochromic WO_3_ supercapacitors in a vertically stacked configuration.^[^ ^101^ ^]^ Through photocharging, the specific areal capacitance, average power density, and energy density of the co‐anode (co‐cathode) device are 286.8 (430.7) F m^−2^, 187.6 (377.0) mW m^−2^, and 13.4 (24.5) mWh m^−2^, respectively (**Figure** **12**A,B). The supercapacitor was fully charged to 0.80 V in 60–70 s of light illumination, followed by 400 s of discharging at a current density of 0.1 mA cm^−2^. The vertical stacking improved the integration level and enhanced the photostability by blocking part of the solar light during the fully charged state. Liu et al. made a power pack integrated with MAPbI_3_ perovskite‐based solar cell and MnO_2_//activated carbon‐based asymmetric supercapacitor on a carbon electrode (Figure 12C).^[^ ^102^ ^]^ The real‐time response of the photovoltage for the power pack with different illuminated areas and their trends of the energy storage proportion during the solar‐charging process are shown in Figure 12D,E. As a result of a larger active area, which can generate a greater photocurrent, the charge time will be significantly reduced, and a higher voltage will be obtained for the capacitor part. When charging starts, the conversion efficiency of the integrated device slowly increases in the beginning and then decreases (Figure 12F). The photocharge/galvanostatic discharge curve in Figure 12G shows the stable performance of the device. The system achieved a stable output voltage of ≈3.8 V within 15 s under the illumination of 1.0 sun. LEDs can be driven after photocharged, but only for several minutes since the device consumes self‐energy [Figure 12H]. The power pack achieved a voltage of 0.84 V under the 1.5G white illumination with an energy storage efficiency of ≈76% and conversion efficiency of 5.26%. Four different photosupercapacitors connected in series achieved overall output efficiency of ≈ 22.99% and demonstrated their great potential in solar energy storage devices and flexible/wearable electronic devices. Similarly, thin and flexible power packs built into a “bambooslip design” on MAPbI_3_‐based solar cells and Co_9_S_8_‐MnO_2_‐based supercapacitors showed promising performance as highly integrated, scalable and flexible photosupercapacitor.^[^ ^103^ ^]^ Recently, Yadav et al. integrated a perovskite solar cell and perovskite supercapacitor based on the same CsPbBr_3_ material into a monolithic structure to avoid internal losses arising in two‐, three‐ or four‐terminal electrode configurations of a photosupercapacitor (Figure 12I).^[^ ^104^ ^]^ The device displayed a good energy storage efficiency of ≈ 87% at an overpotential of 0.8 V and a photoelectrochemical efficiency of ≈ 5.13%.

**Figure 13:**
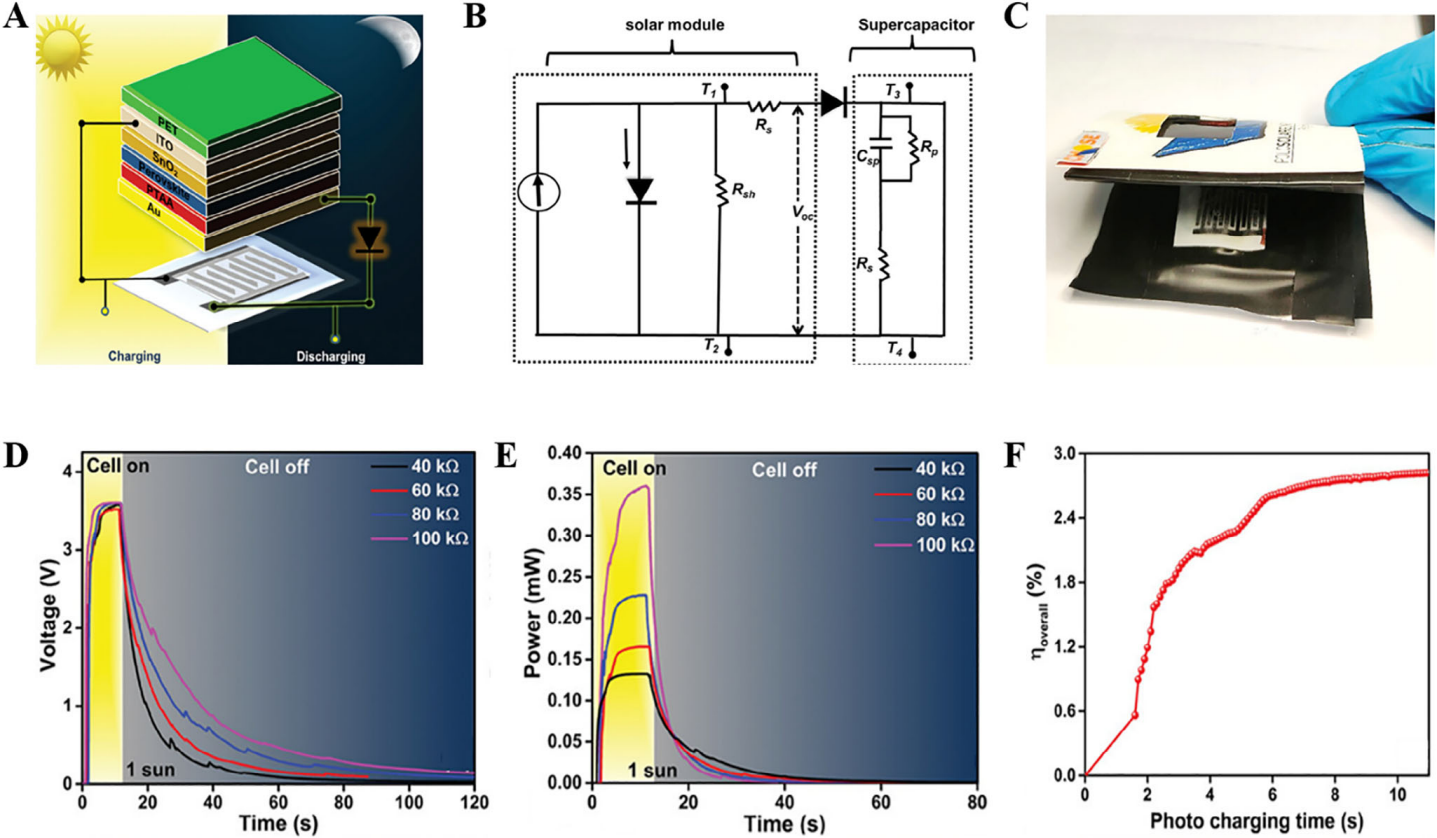
A‐B) Schematic representation and equivalent electrical circuit of the integrated device C) side view shows the stacking of the flexible supercapacitors and the compact integration with the perovskite mini‐module D) The voltage transients of the capacitor during photo‐charging under 1‐sun illumination and galvanostatic self‐discharge at different resistances E) maximum power variation with respect to time under 1‐sun F) overall efficiency variation with photo‐charging time, under indoor light illuminations. Adapted with permission.^[^ ^3^ ^]^ Copyright 2024, Wiley.

By considering the compactness, portability, and ability to power miniaturized embedded electronic and internet‐related devices, micro supercapacitor‐based energy storage systems can be a good choice for integration in photosupercapacitor devices.^[^ ^9^, ^105^ ^]^ By keeping this in mind, Lomeri et al. fabricated an all‐flexible photosupercapacitor mini‐module with a carbon‐based in‐plane supercapacitor and MAPbI_3_‐based solar cell on a paper substrate (**Figure** **13**A–C).^[^ ^3^ ^]^ The maximum overall efficiency of ≈2.8% for the photosupercapacitor under 1‐sun illumination was achieved with an unprecedented wide‐range potential window of 3.8 V, as shown in Figure 13D. The device shows a power density of 0.35 mW cm^−2^ at 100 kΩ of constant load, a maximum overall efficiency of 2.8%, and a storage efficiency of 23% (Figure 13D,E). These results show the device efficiently generates charges with constant performance in steady‐state operation. This was attributed to the flexible architecture of the storage unit (planar supercapacitor) and the conversion unit (perovskite solar mini‐module) and their effective integration. **Table** **2** summarizes the overall performance of reported photosupercapacitors with different perovskite‐based solar cells and supercapacitor electrodes. ^[^ ^32^, ^98^, ^106^, ^107^ ^]^

**Table 2:** Overall performance of photosupercapacitors based on halide perovskites along with their solar cells and supercapacitor components.

Active Perovskite material of the solar cell component	Active electrode material of the Supercapacitor component	Overall performance of the Photosupercapacitor (Overall efficiency = η_ov_ Storage efficiency = η_st_)	Refs.
MAPbI_3‐x_Cl_x_	Graphene	V_oc_ = 0.75 V;	[98]
MAPbI_3_	Polypyrrole	V_oc_ = 1.45 V; η_ov_ = 10%	[99]
MAPbI_3_	PEDOT‐Carbon	η_ov_ = 4.7%; η_s_ **_t_** = 73.77%	[100]
MAPbI_3‐x_Cl_x_	WO_3_	η_ov co‐anode (co‐cathode)_ = 8.25% (11.89%)	[101]
MAPbI_3_	MnO_2_ and porous carbon	V_oc_ = 0.84 V; η_ov_ = 5.26%; η_s_ **_t_** = 76%	[102]
MAPbI_3_	Co_9_S_8_‐MnO_2_	V_oc_ = 0.81 V; η_ov_ = 4.9%; η_st_ = 80%	[103]
CsPbBr_3_	CsPbBr_3_	V_oc_ = 0.8 V; η_ov_ = 5.13%; η_st_ = 87%	[104]
MAPbBr_3_	Carbon	V_window_ = 03.8 V for the power pack; η_ov_ = 2.8%	[3]
MAPbI_3_	Carbon	V_oc_ = 0.91 V; η_ov_ = 7.1%	[106]
MAPbI_3_	NiCo_2_O_4_	η_ov_ = 4.08%	[107]
FA_0.75_Cs_0.25_Pb(I_0.8_Br_0.2_)_3_	N‐doped carbon sphere	V_oc_ = 1.0 V; η_ov_ = 11.5%; η_st_ = 92%	[32]

## 4 Limitations and Progress Strategies of Perovskite‐Based Supercapacitors and Photosupercapacitors

Energy storage technologies, including capacitors, supercapacitors, and emerging photosupercapacitors, demand electrode materials that exhibit high charge storage capacity, long‐term electrochemical stability, and robust structural durability.^[^ ^10^, ^11^ ^]^ Among the various material classes investigated, halide perovskites and perovskite‐based hybrid materials have garnered significant attention due to their tunable optoelectronic properties, high dielectric constants, and ease of processing. However, despite their potential, halide perovskites face critical limitations that hinder their practical application in energy storage systems.

One of the primary challenges lies in their inherent thermal instability, particularly in hybrid organic–inorganic perovskites, where volatile organic cations (e.g., methylammonium or formamidinium) are susceptible to decomposition at elevated temperatures commonly encountered during device operation.^[^ ^108^ ^]^ Furthermore, these materials exhibit pronounced sensitivity to ultraviolet (UV) radiation, which can induce photo‐degradation of the perovskite lattice, resulting in phase segregation and a gradual decline in device performance.^[^ ^109^ ^]^ Another major concern is the hygroscopic nature of many halide perovskites, making them vulnerable to moisture‐induced degradation.^[^ ^110^ ^]^ The ingress of water molecules into the perovskite crystal lattice, leading to irreversible structural breakdown and significant loss in electrochemical functionality. Yet, a critical factor impacting the long‐term stability of halide‐based devices is ion migration, which leads to hysteresis in current‐voltage behavior and performance inconsistency. This is attributed to the high ionic mobility within the perovskite lattice, affecting internal charge distribution and electric fields.

To mitigate these issues and extend the operational lifetime of perovskite‐based energy devices, several material engineering strategies have been proposed. These include compositional tuning, such as partial or complete substitution of volatile organic cations with more stable inorganic counterparts (e.g., Cs⁺), ligand passivation to reduce surface defect densities, encapsulation techniques to physically isolate the perovskite layer from environmental stressors, and morphological optimization through control of particle size and crystallinity to enhance mechanical and chemical resilience. Strategies such as dopant incorporation and grain boundary engineering have been explored to suppress ion migration.^[^ ^111^, ^112^, ^113^, ^114^, ^115^, ^116^ ^]^ Mitigating hysteresis is essential for ensuring the reliability of perovskite‐based energy storage systems. Collectively, these approaches aim to fortify the structural integrity of perovskite materials and enable their reliable integration into next‐generation, high‐performance energy storage systems.

### 4.1 Progress Strategies for Supercapacitors

As discussed about the performance of symmetric electrochemical supercapacitor based on MAPbI_3_ perovskite by Zhou et al. To improve the long‐term stability of halide perovskite materials in electrochemical energy storage applications, the group has employed several strategic approaches.^[^ ^50^ ^]^ Electrolyte engineering plays a crucial role; selecting low‐reactivity or “poor” solvents minimizes the dissolution of perovskite constituents, thereby preserving the structural and chemical integrity of the film. Additionally, incorporating pre‐dissolved ionic species (such as MA^+^ and I^−^) in the electrolyte can help maintain ionic equilibrium and suppress degradation at grain boundaries. Device architecture optimization, including the use of ion‐permeable membranes and symmetric electrode configurations, can effectively decouple electronic conduction from ionic transport, reducing undesirable side reactions. Furthermore, maintaining film integrity through controlled thickness, crystallinity, and surface morphology helps limit ion migration‐induced degradation.^[^ ^50^ ^]^ In another study mentioned previously, Rao et al. have strategies like incorporating spacer cations, compositing the perovskite material, and optimized the precursor concentrations and annealing temperatures.^[^ ^54^ ^]^ Here a quasi‐2D Ruddlesden–Popper perovskite structure was employed, incorporating bulky organic spacer cations like 4‐fluorobenzylammonium (4‐FBA), which improved both thermal and moisture stability due to their hydrophobic nature. The optimized precursor concentration and annealing temperatures promoted vertical crystal growth and high film crystallinity, leading to better morphological integrity. Additionally, compositing the perovskite with acetylene carbon black further improved electrical conductivity and reduced charge transfer resistance, contributing to enhanced electrochemical stability and prolonged cycling performance.^[^ ^54^ ^]^

To enhance the material stability of the Cs_3_Bi_2_I₉‐based supercapacitor, Adams et al. have employed a combination of solution‐processable fabrication, optimized electrode composition, and structural reinforcement techniques.^[^ ^74^ ^]^ The PTFE was used to enhance the mechanical integrity of the electrode film. Moreover, the formation of highly crystalline Cs_3_Bi_2_I₉ coatings with uniform morphology on carbon cloth substrates contributed to stable electrochemical behavior. These strategies collectively enabled excellent long‐term performance, with the device retaining 88% of its initial capacitance after 5000 charge–discharge cycles.^[^ ^74^ ^]^ To study the effect of compositional engineering, Yasmeen et al. employed halide engineering by partially substituting Br^−^ with Cl^−^ in CsSnBr_3_ to form moisture‐stable CsSnBr_2_Cl nanoparticles.^[^ ^77^ ^]^ This modification preserved the structural and electronic integrity of the material even after 75 days of water immersion. In a symmetric two‐electrode configuration using HCl and Na_2_SO_4_ electrolytes, the CsSnBr_2_Cl nanoparticles retained 93% of their capacitance after 5000 charge‐discharge cycles in HCl(aq.), maintaining 91% Coulombic efficiency. The nanoparticles also demonstrated stable operation across a temperature range of 3 to 60 °C.^[^ ^77^ ^]^ In addition to the widely explored strategies such as compositional tuning, electrolyte engineering, and morphology control, several other approaches have shown promise in enhancing the long‐term stability of perovskite‐based supercapacitors. Defect passivation, using small molecules or polymers can effectively reduce trap states and suppress ion migration.^[^ ^117^ ^]^ Interface engineering, through surface treatments or interfacial layers, helps stabilize the electrode–electrolyte boundary and prevent interfacial degradation.^[^ ^118^ ^]^ Encapsulation techniques, using polymeric or inorganic barrier layers protect the perovskite from moisture, oxygen, and UV exposure, thereby extending device lifespan.^[^ ^119^ ^]^ Collectively, these strategies contribute to the enhanced durability and operational reliability of perovskite‐based supercapacitor systems, paving the way for their practical application in next‐generation energy storage technologies.

### 4.2 Progress Strategies for Photosupercapacitors

To explore the stability of the perovskite based photosupercapacitor, Zhu et al. have employed various strategies. A primary approach was the integration of all‐inorganic perovskites, specifically CsPbBr_3_, which are known to exhibit superior thermal and environmental stability compared to their hybrid organic‐inorganic counterparts.^[^ ^91^ ^]^ Furthermore, the device architecture incorporated a protective carbon layer to shield the perovskite material from moisture and oxygen ingress, both of which are significant contributors to perovskite degradation. The use of carbon‐based electrodes not only improved stability but also supported better charge transport and device longevity. Additionally, encapsulation techniques by using simple PET films were used to further isolate the active materials from environmental stressors, ensuring sustained performance over extended operational periods.^[^ ^91^ ^]^ In another study, to enhance the stability by means of structural strategies of electrodes, Zhou et al. employed a combination of material and structural strategies.^[^ ^101^ ^]^ The use of a vertically integrated architecture combining a semitransparent perovskite solar cell (PSC) and a WO_3_‐based electrochromic supercapacitor not only enabled energy harvesting and storage but also improved photostability. A transparent MoO_3_/Au/MoO_3_ (MAM) electrode was used to facilitate light transmission while providing a robust electrical interface. The WO_3_ electrochromic layer acted as a “solar shelter,” modulating its optical transmittance upon charging, which in turn shielded the PSC from prolonged light exposure. This dynamic light filtering effect reduced photodegradation of the PSC and prolonged its operational lifetime under continuous illumination. Additionally, the MoO_3_/WO_3_ counter electrode served as an efficient charge‐balancing component, eliminating the need for a reverse bias during discharging, thus further improving device durability.

To enhance the stability of the integrated PSC and SC system, Liu et al. employed several key strategies.^[^ ^102^ ^]^ A printable carbon electrode was used as a common interface for both the PSC and SC, offering robust chemical stability and preventing degradation typically associated with metal electrodes. The CH_3_NH_3_PbI_3_ perovskite layer was fabricated using a two‐step sequential method, yielding uniform, dense films with large grain sizes, which contribute to improved environmental resistance. The device architecture ensured strong adhesion between layers and minimized internal resistance, helping to reduce self‐discharge. Furthermore, the MnO_2_/carbon composite electrode structure in the SC enhanced ion diffusion and maintained a stable capacitance over 5000 cycles with 96.2% retention. These combined material and structural approaches significantly improved the operational durability and performance consistency of the hybrid energy device.^[^ ^102^ ^]^ In addition to the strategies discussed above, several other approaches can further enhance the stability of perovskite‐based photosupercapacitors. Surface passivation techniques, such as incorporating 2D perovskite layers or hydrophobic polymers, can effectively suppress defect‐mediated degradation and moisture ingress.^[^ ^120^ ^]^ Encapsulation using flexible, UV‐resistant barrier films like parylene or polyimide can offer long‐term environmental protection.^[^ ^121^ ^]^ Doping of perovskite layers with ions such as alkali metals (e.g., Cs^+^, Rb^+^) or halides (e.g., Cl^−^, F^−^) has been shown to improve crystallinity and suppress ion migration, thereby enhancing thermal and operational stability.^[^ ^122^ ^]^ Additionally, interface engineering, such as introducing interlayers like NiO, TiO_2_, or graphene oxide—can mitigate interfacial recombination and promote charge extraction, further improving device robustness. These advanced design strategies, in conjunction with optimized architectures, hold great promise for realizing durable, high‐performance perovskite‐based integrated energy devices.

## 5 Conclusion and Future Perspectives

**Figure 14:**
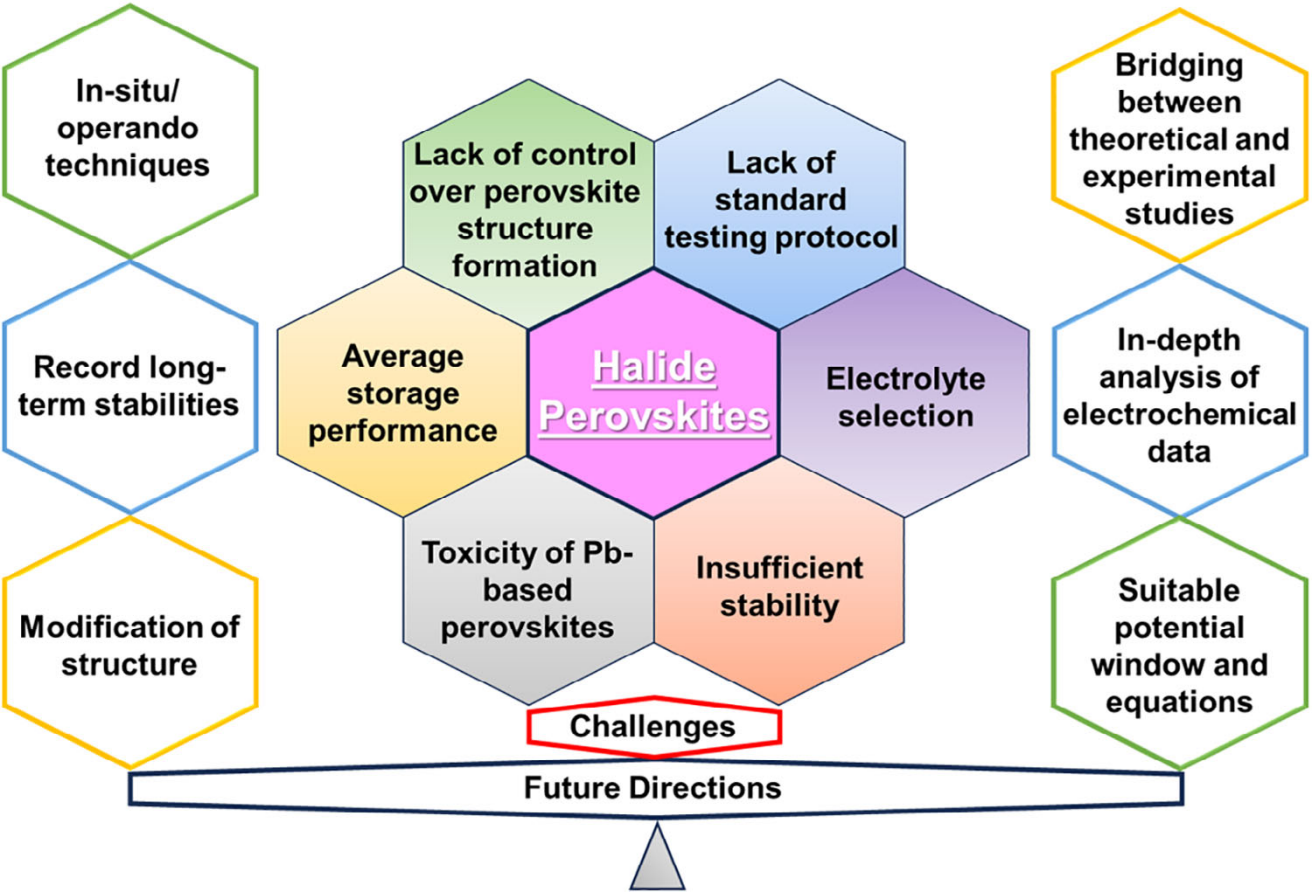
Challenges and future perspectives presented for halide perovskite development in supercapacitor field.

Because of their good electrical conductivity, good ionic conductivity, high crystallinity, good charge storage capacity and electrochemical properties, facile preparation and cost‐effectiveness, halide perovskites have gained attention as crucial materials for supercapacitor and photosupercapacitor devices. Further tuning of their properties by doping in cation or anion sites, or creation of defects/vacancies, promotes its application for energy storage applications since it facilitates ionic migration, ionic conductivity, and surface area. This review addresses the advantages of halide perovskites in the field of supercapacitors and photosupercapacitors, and recent developments in this research field are discussed. Halide perovskite‐based photosupercapacitors displayed promising outcomes in terms of overall efficiency and storage efficiency, which are the highest values reported so far. However, instability and toxicity remain the main worries for their possible commercialization in future, which need to be addressed (**Figure** **14**). By considering the toxicity, environmental and health risks due to Pb, further research on Pb‐free, partially, or fully Pb‐substituted with non‐toxic metals such as Sn, Ge, Bi, Sb, etc., based halide perovskites needs to be explored for supercapacitor applications. The structure of perovskite is very sensitive to ambient conditions such as moisture, temperature, and light. Halide perovskites can degrade in humid environments due to water molecules soaking through their lattices, and they lose their original structure. Even at low temperatures, halide perovskites react with water vapour to produce lead iodide as a byproduct. The organic part (such as methylammonium) of the halide perovskite structure decomposes under heat treatment, which impacts its structural stability and optoelectronic capabilities. The structure of perovskite gets damaged by UV light exposure, which can also cause defects inside it. Also, the stability and performance of the electrochemical supercapacitor are significantly impacted by ion migration during charging/discharging and phase transition of the perovskite structure. These different parameters change the composition and phase of the perovskite structure, which greatly affects the stability of the perovskite structure. It is very important to preserve perovskite's performance from synthesis to application for commercialization purposes. It is difficult to prepare repetitively uniform‐sized, large‐scale perovskites with the required specifications. Therefore, the operating lifetime and scalability of halide perovskite‐based devices are significantly limited by these stability problems.

Further research in this field by exploring newly emerging perovskites and their modification by different engineering tactics such as doping, defect/vacancy engineering, heterostructure/hybrid engineering, phase, facet, and morphology engineering, tuning of dimensionality, integration strategies, configuration design, etc. will be helpful to achieve better performance. These types of modifications substantially impact the storage capacity, efficiency, and cyclic stability of the device by increasing the availability of active sites for ion storage, impacting ion transport, and ion diffusion. These strategies will help to solve the issue of stability, reproducibility, and better energy storage performance. Since the selection of appropriate electrolytes plays an important role in designing supercapacitors with high working windows, energy density, and stability, further research in this direction needs to be carried out. The field of hybrid capacitors, supercapattery, micro supercapacitors, electrochromic supercapacitors, self‐healing supercapacitors, and integrated supercapacitors with other functionalities such as photodetection, gas sensing, biosensing, etc. for the halide‐based materials are either in the preliminary stage or not explored yet. By considering the important properties of the halide perovskites, they can be considered for the fabrication of these smart, hybrid, and integrated devices for future electronics. Scalability and integration into wearable technology, sustainable production methods and flexible electronics based on the halide perovskites could make them ideal candidates for future commercialization. A variety of structures can be developed for halide perovskites. The performance of photosupercapacitors is determined by a number of interrelated factors, including energy storage capacity, charge transfer, and light absorption. Advanced techniques, like machine learning (ML) and artificial intelligence (AI) can help to predict application‐suitable compositions of structures. Machine learning has emerged as a powerful and essential approach for accelerating the discovery and optimization of new perovskite materials. By enabling high‐throughput computational screening across vast compositional spaces, machine learning significantly reduces the need for exhaustive experimental trials. Through the analysis of large‐scale datasets, these algorithms can uncover hidden correlations between structural features and performance metrics, thereby guiding the design of perovskite compositions with improved efficiency and long‐term stability. Notably, machine learning models can predict how different perovskite formulations respond to environmental stressors, offering valuable insights for developing commercially viable and durable perovskite‐based energy devices. These tools will also aid in the comprehension of charge dynamics, photoconversion efficiency, optimised energy alignment, and device energy/power density. Theoretical works on the important properties and energy storage capabilities of halide perovskites should be carried out for the design of high‐performance supercapacitors and to understand their charge storage mechanisms. Integration of various in‐situ/operando spectroscopic characterizations such as in‐situ XRD, in‐situ XPS, in‐situ Raman spectroscopy and in‐situ atomic force microscopy (AFM) etc. with electrochemical performance studies should be focused for the understanding of the charge storage mechanisms and to gain insights on the degradation processes of the halide‐based perovskite supercapacitors, which will be helpful to fabricate more efficient devices and energy storage systems.

## Conflict of Interest

The authors declare no conflict of interest.

## Data Availability

No primary research results, software or code have been included, and no new data were generated or analysed as part of this review.
